# Involvement of the GABAergic system in PTSD and its therapeutic significance

**DOI:** 10.3389/fnmol.2023.1052288

**Published:** 2023-02-01

**Authors:** Junhui Huang, Fei Xu, Liping Yang, Lina Tuolihong, Xiaoyu Wang, Zibo Du, Yiqi Zhang, Xuanlin Yin, Yingjun Li, Kangrong Lu, Wanshan Wang

**Affiliations:** ^1^Southern Medical University, Guangzhou, China; ^2^Department of Applied Psychology of School of Public Health, Southern Medical University, Guangzhou, China; ^3^Department of Basic Medical of Basic Medical College, Southern Medical University, Guangzhou, China; ^4^Eight-Year Master’s and Doctoral Program in Clinical Medicine of the First Clinical Medical College, Southern Medical University, Guangzhou, China; ^5^Department of Medical Laboratory Science, School of Laboratory Medicine and Biotechnology, Southern Medical University, Guangzhou, China; ^6^Guangdong Provincial Key Laboratory of Construction and Detection in Tissue Engineering, Southern Medical University, Guangzhou, China; ^7^Department of Laboratory Animal Center, Southern Medical University, Guangzhou, China

**Keywords:** post-traumatic stress disorder, GABA, fear memory, amygdala, neurotransmitter

## Abstract

The neurobiological mechanism of post-traumatic stress disorder (PTSD) is poorly understood. The inhibition of GABA neurons, especially in the amygdala, is crucial for the precise regulation of the consolidation, expression, and extinction of fear conditioning. The GABAergic system is involved in the pathophysiological process of PTSD, with several studies demonstrating that the function of the GABAergic system decreases in PTSD patients. This paper reviews the preclinical and clinical studies, neuroimaging techniques, and pharmacological studies of the GABAergic system in PTSD and summarizes the role of the GABAergic system in PTSD. Understanding the role of the GABAergic system in PTSD and searching for new drug targets will be helpful in the treatment of PTSD.

## 1. Introduction

Post-traumatic stress disorder (PTSD) refers to a delayed and persistent mental disorder that is caused by an individual’s experience of a sudden and traumatic event, such as war, earthquake, car accident, sexual assault, or exposure to extreme stress (Battle, 2013). More than 70% of adults worldwide have experienced at least one traumatic event in their lifetime, and 31% have experienced four or more (Benjet et al., 2016). The lifetime prevalence of PTSD varies by social background and country of residence, ranging from 1.3 to 12.2%, and the 1-year prevalence ranges from 0.2 to 3.8% (Karam et al., 2014). As PTSD was originally thought to be a physiological disorder rather than a mental one, some early studies used physiological methods to explore the physical abnormalities of patients, such as heart rate, skin conductance, and facial electromyography (EMG). These measures have been widely used in PTSD studies and have strongly demonstrated high emotional responses to trauma-related cues and excessive startle responses (Vasterling and Brewin, 2005). One of the earliest and most common findings of PTSD studies is the autonomic reactivity of patients to traumatic stimuli (e.g., heart rate and skin conductance) and facial EMG. Studies have demonstrated that the response to trauma-related cues is related to the severity of the disease (Wilson and Keane, 1997; Wolfe et al., 2000; Suendermann et al., 2010). In addition, the exaggerated startle response of PTSD patients has been documented in numerous blinks and electromyogram measurements (Orr et al., 1997, 2003; Pole, 2007; Pole et al., 2009). In mammalian studies (Koch and Schnitzler, 1997), the acoustic startle response may be a valuable model for studying the general principles of sensorimotor-motivational information processing at the behavioral and neurophysiological levels. Besides, studies of acoustic startle responses in rodents have shown that phasic fear is mediated by the amygdala, which sends outputs to the hypothalamus and brainstem to produce fear symptoms (Davis et al., 2010). However, it is still not clear whether this represents an increased neurological sensitivity to situational threats in people with PTSD. According to the latest Diagnostic and Statistical Manual of Mental Disorders by the American Psychiatric Association (DSM-5, 5th edition) (Battle, 2013) and the International Classification of Diseases, 11th Edition (ICD-11) published by the World Health Organization (Almeida et al., 2020), the core features of PTSD include intrusive symptoms, avoidance symptoms, and excessive alertness. According to ICD-11, these three symptom groups are also part of the complex post-traumatic stress disorder (CPTSD) diagnosis. Additionally, In CPTSD three other symptom groups can be summarized as disturbances in self-organization: Emotion regulation difficulties, relationship difficulties, and negative self-concept (Maercker, 2021). The DSM-5 also emphasizes cognitive and emotional changes, and patients may experience cognitive decline, depression, loss of interest, indifference, and other manifestations. The clinical diagnosis of PTSD is established when these symptoms persist for more than 1 month.

At present, the neurobiological mechanism of PTSD has not been confirmed, and the research directions mainly include four aspects: (1) Genes involved with monoamine and the hypothalamic-pituitary-adrenal (HPA) axis function have been examined extensively in epigenetic and genetic studies of PTSD risk and separately in studies of disease risk and response to treatments for mood disorders (Kato and Serretti, 2010; Domschke et al., 2014; Zannas et al., 2015; Smoller, 2016). Two of the most commonly characterized genes in this regard are the serotonin transporter (SLC6A4) and FK506 binding protein 5 (FKBP5) (Bishop et al., 2021); (2) neuroendocrine dysfunction, such as the increased secretion of catecholamines (Olson et al., 2011) and decreased secretion of 5-hydroxytryptamine (5-HT) hormones (Liu et al., 2018) and corticosterone (Geracioti et al., 2008); (3) changes in the neural structure and circuitry. Basic and clinical studies have demonstrated that structural and functional abnormalities in the hippocampus, prefrontal cortex (PFC), amygdala, and other brain areas were observed in both animal models and individuals with PTSD (Rauch et al., 2006; Hayes et al., 2012; Disner et al., 2018).

Gamma-aminobutyric acid (GABA) is an important inhibitory neurotransmitter in the central nervous system (CNS), which can reduce neuronal activity, prevent nerve cells from overheating, and calm nerves. Besides, GABA is also an active amino acid that plays an important role in the process of energy metabolism in the human brain. For example, GABA participates in the tricarboxylic acid cycle in the brain and promotes the metabolism of brain cells. At the same time, GABA can also improve the activity of glucose phosphatase during glucose metabolism, increase the generation of acetylcholine, expand blood vessels to increase blood flow, and reduce blood ammonia to promote brain metabolism and restore the function of brain cells (Petroff, 2002). As one of the inhibitory neurotransmitters of the CNS, GABA plays an essential role in regulating the stress response, emotion, and registration and encoding of fear memory (Corcoran and Maren, 2001). Dysfunction of the GABAergic system has been proven to be one of the mechanisms of PTSD, with several studies demonstrating that PTSD can reduce the levels of GABA and its receptors in some brain regions. Positron emission tomography (PET) was used to identify post-war PTSD patients, and it was observed that the distribution of benzodiazepine-GABA receptors decreased in the PFC, as well as the entire cortex, hippocampus, and thalamus (Geuze et al., 2008). Other studies have shown that the levels of GABA in the occipital and temporal lobes of PTSD patients were significantly decreased compared to controls, resulting in sleep disorders (Meyerhoff et al., 2014). Studies on animals have shown that the anxiety and fear behaviors of mouse models of PTSD improved after the administration of exogenous tetrahydroprogesteroneal, which may be induced by the enhancement of GABA function mediated by allopregnanolone (Evans et al., 2012).

Recent preclinical and clinical data indicate that GABA, which is a major inhibitory neurotransmitter involved in the pathophysiology of PTSD, plays an important role in stress. Changes in the GABA system are related to the pathogenesis of PTSD. Understanding the systemic changes in GABA in PTSD will not only contribute to the diagnosis of PTSD but also reveal new targets for pharmacological intervention.

In this review, we review the role of the GABAergic system from the phenomenon to the mechanism, and then to the clinical guidance in PTSD. We discuss fear memory, an important component of PTSD, and the role of GABA receptors in fear memory formation and extinction. By reviewing the changes in the GABA system in different brain regions in PTSD, we state the ubiquitous and heterogeneous nature of GABA in the brain. Furthermore, the role of GABA in PTSD and its mechanism were further discussed, which involved how the GABA system interacts with other systems, including the HPA axis and the endocannabinoid system (ECS), as well as the role of glutamate (Glu) and GABA signal imbalance in the brain in PTSD. Finally, we bring together the clinical, preclinical and neuroimaging evidence of changes in the GABA system in PTSD, as well as GABA mechanisms of several clinical drugs. We treat the “GABAergic system” as a single unified neurotransmitter system. It is useful to highlight large-scale, non-specific changes in GABA signaling to establish the importance of dysregulation of GABA function in PTSD.

## 2. GABAergic system and PTSD

**FIGURE 1:**
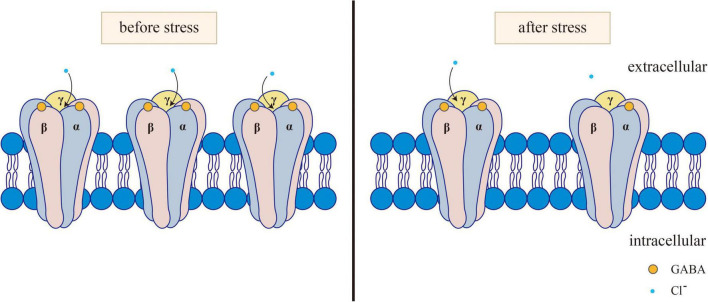
Changes of GABAA receptor before and after stress. Normally, GABA activates the opening of GABAA receptors. After the GABAA receptor is activated, it can selectively let Cl^–^ through, causing the hyperpolarization of neurons. After stress, GABA levels and GABA-A receptors decrease, and GABA binding to GABA-A receptors subsequently decreases, resulting in reduced Cl- influx.

The GABAergic system plays a certain role in the onset of depression, anxiety, and other mental disorders. Clinical and preclinical studies have shown that the inhibitory effect of the GABAergic system in anxiety patients is reduced (Domschke and Zwanzger, 2008). The receptor of GABA refers to the part of the postsynaptic membrane that can recognize and bind GABA. When it binds to GABA, it can cause changes in membrane ion permeability. The expression or dysfunction of GABA receptors is associated with mental illness. Three main subtypes of GABA receptors, namely, GABA-A, GABA-B, and GABA-C, have been identified so far. Among them, GABA-A and GABA-C receptors are ligand-gated ion channels. The transmembrane receptor GABA-B binds with the G protein to activate the second messenger system (Chebib and Johnston, 1999). GABA-A receptors are widely distributed throughout the nervous system and peripheral tissues. GABA-B receptors are found in the olfactory bulb, neocortex, hippocampus, thalamus and cerebellum of mammals (Lujan and Ciruela, 2012). GABA-C receptors are found mainly in the retina, which is also distributed in the spinal cord, thalamus, pituitary gland, and intestine of mammals (Zhang et al., 2001). The rapid inhibition of the neurotransmitter GABA is mediated by GABA-A receptors. Furthermore, various subtypes of the GABA-A receptor have been identified, including α1–6, β1–3, γ1–3, δ, ε1–3, θ, and π (Jacob et al., 2008). The main receptors mediating neural inhibition in the brain are GABA-A receptors, and changes in the expression or function of these receptors in patients are increasingly related to the etiology of anxiety and depression (Merali et al., 2004; Sanacora et al., 2004; Bhagwagar et al., 2008; Poulter et al., 2008; Klempan et al., 2009; Sequeira et al., 2009; Craddock et al., 2010; Levinson et al., 2010; Figure 1). In particular, the changes in GABA-A receptor subunits play an important role in the amygdala and PFC mediating fear memory, as detailed in section “2.1.2. Effects of the GABAergic system on fear memory in specific brain regions.” In addition, decreased levels of GABA are associated with stress responses (Dolfen et al., 2021), and GABA transmitters mediate stress and fear responses mainly by binding to their receptors.

### 2.1. Fear memory

**FIGURE 2:**
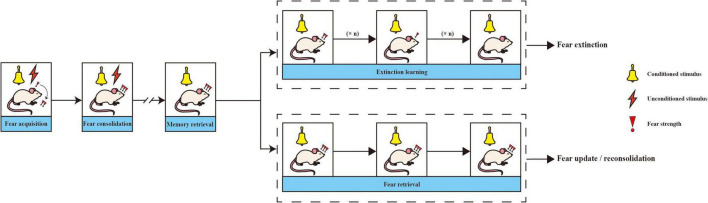
The process of fear memory. When a neutral cue or context [a sound, light, or conditioned stimulus (CS)] occurs with a highly aversive signal or unconditioned stimuli (US) and leads to fear behaviors or conditioned fear responses (CRs), fear acquisition arises. Fear memory will be consolidated within a few hours to a day of fear acquisition. When CS occurs alone, the animal will retrieve the fear memory and lead to CR. After that, if CS has been presented alone without US, fear memory becomes unstable and two different processes could occur. One is called fear extinction, which occurs after CS appears alone repeatedly many times. As a result, fear strength will decrease. The other is reconsolidation, where the fear the memory can be updated or left intact.

Generally, PTSD is regarded as a high fear response to a threat. Thus, fear is an important target in the neurobiology of PTSD. The process of fear memory is divided into several stages, including fear acquisition, fear consolidation, fear destabilization/reconsolidation and fear extinction (Liberzon and Abelson, 2016; Figure 2).

Classical (or Pavlovian) fear conditioning is an effective behavioral paradigm for studying the mechanisms of associative fear learning and memory processes. In rodents, fear indicators are typically assessed by freezing behaviors (Bouton and Bolles, 1980), which is characterized by immobility and the absence of any movement except for breathing. Freezing is a potent conditioned fear response in rats and mice. And it has a great advantage over many other fear measures because freezing is not a typical response of rats or mice to ordinary new stimuli (Anagnostaras et al., 2010). A computerized method based on latency between photobeam interruption measures is used as a reliable scoring criterion in mice, which can reduce bias or inconsistencies (Valentinuzzi et al., 1998). It is important to note that when testing different strains of mice, it would be essential to validate the testing or scoring procedure (Valentinuzzi et al., 1998). To avoid artificially inflated lighting sources and thus ensure freezing behavior is induced in a well-controlled manner, it is suggested to report Lux and control the lighting properly (Neuwirth et al., 2022). When a neutral cue or context [a sound, light, or conditioned stimulus (CS)] occurs with a highly aversive signal or unconditioned stimuli (US) and leads to fear behaviors or conditioned fear responses (CRs), which include freezing, a systemic response to behavior besides breathing, fear acquisition arises [47]. The training box used in the Pavlov paradigm itself can be used as an environmental situational stimulus (CS), which is matched with an aversive plantar shock (US) multiple times. Animals learn the connection between the CS and the US and obtain the fear response (CR) to the environment. This process is called contextual fear conditioning (CFC). If a cue [usually an auditory stimulus (CS)] is matched with a foot shock (US) multiple times during the training, the auditory stimulus (CS) will be separated from the environment and associated with the foot shock (US) to form the CR to the auditory stimulus (CS). This process is called cued fear conditioning (Maren et al., 2001). The exposure of an individual to CS stimulation is sufficient to trigger a fear response (CR). This process is called retrieval of fear memory (Pape and Pare, 2010). Following retrieval, previously formed memory is destabilized, which is similar to the unstable state when the memory has just been acquired, and requires new protein synthesis to restabilize, a process referred to as reconsolidation. Reconsolidation acts to stabilize, update, or integrate new information into long-term memories (Izquierdo et al., 2016). Memory-dependent reconsolidation modification may serve as a therapeutic target to modulate the enhanced fear response commonly associated with debilitating mental disorders. People with PTSD are unable to modify or weaken memories through the reconsolidation process (Ferrara et al., 2019). Once the animals have acquired a CR, repetition of the CS alone without the US usually reduces the CR: this is called extinction. Fear extinction indicates the formation of a new competing memory, which is similar to fear conditioning, rather than the elimination of the original fear memory. In this process, the organism is enabled to acquire an association between CS and no-US, which competes with the conditioned fear memory (Bouton and Bolles, 1980). It is known that extinction generates a new memory engram, so it can be regarded as a process of active learning (Maddox et al., 2019).

#### 2.1.1. The interplay between GABA and fear memory

Exposure to trauma can not only damage the body’s physiological and psychological adaptation to stress (Lissek and van Meurs, 2015; Hill et al., 2018) but also alter to the formation and consolidation of associative fear memories (Parsons and Ressler, 2013; Elms et al., 2019). Frequent exposure to fear-related cues can impact the initial consolidation and subsequent retrieval of memory (Maddox et al., 2019), causing anxiety and trauma-related ailments such as PTSD. The persistent presence of negative cognition will impair the inhibition of invasive memory (Meiser-Stedman et al., 2009; Catarino et al., 2015). Thus, memories related to traumatic events will be repeated, while frequent flashbacks and avoidance behaviors will, in turn, worsen negative cognition, resulting in anxiety and depressive symptoms. Thus, a vicious cycle is formed.

Fear conditioning is a highly conservative form of emotional learning that occurs when an environmental stimulus predicts aversive events. This type of learning allows a previously neutral stimulus to trigger a fear response that prepares the animal for the threat and helps it escape (LeDoux et al., 1988; Blanchard and Blanchard, 1989; Fendt and Fanselow, 1999; Babaev et al., 2018). Neural circuits and cellular mechanisms that mediate fear conditioning have been extensively described, among which the inhibitory regulation of GABA neurons is crucial for the precise regulation of the consolidation, expression, and extinction of fear conditioning (Fendt and Fanselow, 1999; Zhang and Cranney, 2008; Makkar et al., 2010). The antagonists of GABA such as bicuculline (Castellano and McGaugh, 1990) have been demonstrated to enhance memory consolidation, while GABA agonists such as muscimol (Akirav et al., 2006) inhibit memory consolidation. These data suggest the involvement of GABA receptors in memory acquisition and consolidation. The role and mechanism of GABA in PTSD and fear memory extinction are still unclear. A study indicated that GABA signals promoted extinction (Berlau and McGaugh, 2006), while another study found that the activation of GABA signals prevented extinction (Singewald et al., 2015). The divergent role of GABA in extinction may be related to the different distribution of GABAergic neurons in the brain, but this still needs further explanation.

#### 2.1.2. Effects of the GABAergic system on fear memory in specific brain regions

**FIGURE 3:**
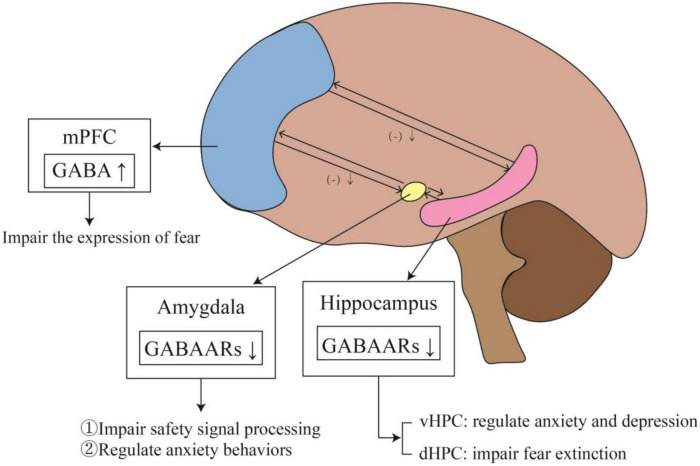
The changes and effects of the GABAergic system in specific brain regions. The outputs of mPFC to the amygdala and hippocampus are inhibitory. When PTSD develops, the level of GABA in mPFC rises, which weakens the inhibition of mPFC to amygdala and hippocampus, affecting fear memory. Meanwhile, elevated GABA level in mPFC impairs fear expression. In addition, the reduction of GABAARs in the amygdala impairs safety signal processing and regulates anxiety behaviors. The expression of GABAARs also decreases in the hippocampus. In vHPC, it leads to anxiety and depression, while in dHPC affected fear extinction.

Most commonly, studies involved in fear memory include the PFC, amygdala and hippocampus. A summary of the changes and effects of the GABAergic system in specific brain regions can be seen in Figure 3. Under normal conditions, the PFC exerts inhibitory control over the amygdala. When the inhibitory effect of the PFC on the amygdala is weakened, it leads to excessive activation of the amygdala. This reduction in top-down control leads to impaired fear extinction. Michels et al. (2014) found increased GABA in the dorsolateral PFC of PTSD patients, indicating an overall shift toward inhibitory tone. Schneider et al. (2016) also showed similar changes in PFC inhibitory tone. GABA was increased in the PFC, but Glu was not changed. This change in GABA may indicate reduced excitatory activity of the PFC. A temporary increase of GABA in PFC can initiate long-term plasticity of the PFC. This change in PFC may affect the activity of the network, transmit through connections to the midline thalamus or entorhinal cortex, and ultimately affect the excitability of the hippocampus (Kyd and Bilkey, 2005). The increased excitatory tone in the hippocampus may lead to impaired fear extinction. The ventral hippocampus (vHPC) can directly project to the PFC and amygdala, affecting their activity (Ishikawa and Nakamura, 2006; Sierra-Mercado et al., 2011). The vHPC projections to the PFC can activate GABAergic neurons in the dorsal PFC and anterior cingulate.

##### 2.1.2.1. Amygdala

Previous studies have used specific agonists of GABA receptors to inhibit the function of specific brain regions, aiming to clarify the role of each brain region in behavior. The amygdala is crucial for fear conditioning and is a key brain structure for the acquisition and expression of conditioned fear responses (LeDoux, 2000; Sah et al., 2003; Chen et al., 2006). The amygdala consists of the basolateral amygdala (BLA) and the central amygdala (CeA). The BLA can be subdivided into the lateral amygdala (LA), basal amygdala (BA), and basal medial amygdala (BMA), while CeA can be further subdivided into lateral CEA (CEl) and medial CEA (CEm) (Tovote et al., 2015). The BLA and the CeA are the main research objects of fear memory, and both receive input from the cortex. The BLA consists of glutamatergic spiny projection neurons (approximately 80%) and GABAergic interneurons (McDonald, 1982; McDonald and Augustine, 1993). In contrast, the CeA is composed of GABAergic medium spiny neurons (Sah et al., 2003). In addition, there is a class of GABAergic neuronuclear nuclei between the BLA and CeA that provide feedforward inhibition signals for the BLA and CeA (Tovote et al., 2015). When the amygdala was inactivated with the GABA-A agonist muscimol, the inactivation of the amygdala resulted in deficits in situational and delayed conditioning (Raybuck and Lattal, 2011). Conversely, hyperactivity of the amygdala may be associated with impaired safety signal processing in PTSD (Christianson et al., 2012). In the classical fear conditioning circuit model, the LA is the main part of the formation and storage association of CS and US (LeDoux, 2000; Maren and Quirk, 2004; Chen et al., 2006; Sigurdsson et al., 2007; Sah et al., 2008). Besides, CeA is involved not only in fear expression but also in the learning and consolidation of Pavlovian fear conditioning (Wilensky et al., 2006). Conditioned fear can activate the inhibitory network in the CeA, inhibit the CeA-periaqueduct gray pathway, and finally promote fear (Ball et al., 2017). Conditioned fear was observed to reduce the frequency of miniature inhibitory postsynaptic current (mIPSC), as well as the expression levels of GABA type-A (GABAAR) γ2 and β2 receptors of the amygdala (Lin et al., 2011). Moreover, α2 receptors in the amygdala are involved in the regulation of anxiety behaviors (Lehner et al., 2010).

##### 2.1.2.2. Hippocampus

The hippocampus plays a key role in the encoding, storage, and retrieval of fear memories (Anagnostaras et al., 1999; Bast et al., 2001). The vHPC inputting directly into prelimbic cortex enables to form contextual fear memory, which is necessary for the subsequent rapid expression of a fear response (Twining et al., 2020). Contextual fear conditioning was impaired after unilateral microinjection of GABAA agonist muscimol into the vHPC (Gilmartin et al., 2012). Another study (Herbst et al., 2022) showed that shock encoding in the vHPC modulated the expression of learned fear in a sex-specific manner, i.e., preventing hippocampal shock-evoked firing led to a more prolonged expression of CS freezing across test trials, an effect observed in males but not in females. Moreover, Almada et al. (2013) reported that the injection of the GABAA agonist muscimol into the dorsal hippocampus (dHPC) of mice impaired the expression of situation-associated fear memory. Similarly, another experiment revealed that the injection of muscimol into the dHPC inhibited the retrieval of short-term fear memory but not long-term fear memory (Raybuck and Lattal, 2014). This may be because the enhanced transmission of GABAergic signals can inhibit excitatory pyramidal neurons and prevent them from participating in the encoding and storage of fear memories. For example, the inhibition of hippocampal CA1 pyramidal neurons can impair the expression of fear memories associated with recent situations (Sakaguchi et al., 2015). In addition, one study showed that after stress exposure, the expression of the α1 and α2 subunits of GABA-A receptors in the vHPC is increased, contributing to stress recovery in rats (Ardi et al., 2016).

Stress and stress hormones such as corticosterone affect long-term potentiation (LTP) differently in the ventral and dorsal hippocampus. Maggio’s study (Maggio and Segal, 2007) found that the magnitude of LTP in the vHPC was significantly smaller than that elicited in the dHPC. However, stress actually enhanced LTP in the vHPC, which is unlike the case in the dHPC. This different property is controlled by a metabolic Glu receptor (Sakaguchi et al., 2015). Glucocorticoid receptor (GR) and mineralocorticoid receptor (MR) antagonists blocked the stress on dHPC and vHPC, respectively. The vHPC and dHPC are distinguished not only by differences in anatomical structure but also by differences in function and gene expression. The vHPC is more involved in anxiety and emotional processing, while the dHPC is more involved in learning and memory (Fanselow and Dong, 2010). People with PTSD may suffer from anxiety and depression, and the neurobiological mechanism of PTSD involves fear learning and fear memory. Therefore, it may be difficult to distinguish the role between the vHPC and dHPC in PTSD, and further research is needed in the future.

##### 2.1.2.3. Prefrontal cortex

Early studies on the connection between the medial prefrontal cortex (mPFC) and fear have been conducted through damage experiments. Morgan and LeDoux (1995) demonstrated that the overall damage to the mPFC, including the anterior cingulate cortex (ACC), prelimbic cortex (PL), and infralimbic cortex (IL), impaired fear acquisition rather than fear extinction. Subsequent studies have shown that damage to PL alone (mainly the dorsomedial region) enhances the acquisition of fear memories and prevents fear extinction (Davidson et al., 2000). The expression of fear was also impaired by the injection of muscimol to reduce the activity of PL neurons before extinction training (Marquis et al., 2007). Another study revealed that specific damage to the ventromedial prefrontal cortex (vmPFC) is necessary for recalling a previously learned extinction memory, rather than learning extinction *per se* (Quirk et al., 2000). These studies suggest that PL is involved in the expression of extinction learning, whereas the vmPFC is in extinction consolidation. Additionally, different subregions of the mPFC play different roles in fear expression, which may be related to differences in the distribution of GABA receptor subtypes.

Moreover, the PFC controls the stress response and participates in the regulation of emotion through inhibitory GABAergic projection to the amygdala (Davidson et al., 2000). The findings of Lu et al. (2017) indicate that early traumatic stress can increase the expression of the α2 subunit in the PFC. Deficiency of GABAAR in the PFC will weaken its control over downstream neuroanatomical regions and increase the expression of the γ2 subunit in the amygdala, leading to defective GABAergic neurotransmission. This fails to erase fear-related memories (Koenigs and Grafman, 2009; Milad et al., 2009) and impairs the control of fear responses in PTSD (Aupperle et al., 2012), thereby increasing susceptibility to PTSD in adulthood.

### 2.2. Changes in the GABAergic system in PTSD

Exposure to war or other traumatic events is associated with a higher risk of mental health problems, including PTSD. The clinical symptoms of these stress-related anxiety disorders are associated with the long-term dysfunction of inhibitory and excitatory neurotransmission and potential neuronal damage (Hageman et al., 2001; Charney, 2004). Both GABA and Glu are important neurotransmitters that are associated with general neurological function, memory registration, and encoding of emotional and fearful memories in anxiety disorders, especially anxiety (Pham et al., 2009). Chronic stress reduces the activity of the neurotransmitter GABA in the hippocampus and PFC, as well as the inhibition of the HPA axis, resulting in anxiety and depression (Mikkelsen et al., 2008). In previous neuroimaging studies, concentrations of these amino acids (in addition to the density and activity of neurotransmitter receptors and transporters) have been associated with mood disorders and memory dysfunction (Krystal et al., 2002). It is suggested that the normal excitation-inhibition balance in the CNS is broken between excitatory glutamatergic neurons and inhibitory GABAergic neurons due to the weakened function of GABAergic neurons, which may lead to the onset of mental disorders. The decrease in plasma GABA levels after trauma can predict the development of PTSD (Vaiva et al., 2004), suggesting that the GABAergic system is involved in the pathophysiology of PTSD. Various studies, including preclinical and clinical studies, neuroimaging techniques, and pharmacological studies, have reported the involvement of the GABAergic system in PTSD.

#### 2.2.1. Preclinical and clinical evidence

Preclinical and clinical data indicate that GABA plays an important role in stress and that changes in the GABAergic system are related to the pathogenesis of PTSD, as it is the main inhibitory neurotransmitter involved in the pathophysiology of PTSD (Shin and Liberzon, 2010; Averill et al., 2017; Ghosal et al., 2017). Increasing evidence has suggested that the overactivation of the amygdala in PTSD patients is associated with a decreased inhibitory function of GABA (Pitman et al., 2012). Decreased inhibitory function of GABA thus plays an important role in the expression of stress-induced fear memory and the production of neuronal LTP. Reduced binding of GABA-A receptors in the cortex, hippocampus, and thalamus has been reported in veterans with PTSD, and the overall inhibitory function of the brain is deficient (Geuze et al., 2008). In victims of traffic accidents that meet the criteria of trauma exposure, a lower level of post-traumatic plasma GABA predicts the development and more chronic course of PTSD (Vaiva et al., 2006). In addition, PET studies have demonstrated that the binding of hippocampal GABAARs to benzodiazepine (BDZ) in PTSD patients is reduced (Geuze et al., 2008; Möller et al., 2016). These studies suggest that the increased expression of GABA-A receptors under stress may contribute to stress adaptation, while decreased expression of GABA-A receptors may contribute to PTSD susceptibility. Consistent with this view, a recent study in the ventral tegmental region reported that the mRNA levels of GABAergic genes were down-regulated in stress-prone mice but not in stress-adapted mice (Sun et al., 2018). Other clinical studies have reported that PTSD patients have impaired recognition of environmental cues, suggesting that impaired hippocampal function may play an important role in the development of PTSD. Exposure to unavoidable foot shock results in reduced GABA-A receptor function, as well as cortical and hippocampal binding (Lippa et al., 1978; Medina et al., 1983; Drugan et al., 1986). In the stress-restress paradigm, an animal model exhibits a similar pattern as PTSD, wherein stress causes a sustained decline in hippocampal GABA levels (Harvey et al., 2004). Animal experiments have demonstrated that chronic stress can reduce the number of GABA neurons in the hippocampal CA1 and CA3 regions (Megahed et al., 2015). Chronic exposure to stress levels of adrenal corticosteroids leads to the abnormal expression of GABA and receptor subunits in the hippocampal CA1 and CA3 regions, although the changes in the expression of different subunits follow different patterns in the CA1 and CA3 regions (Orchinik et al., 1995). Moreover, the expression of the GABAA α2 and γ2 receptors in the PFC of adult rats decreased after shock stress in childhood, indicating that traumatic stress may reduce the activity of GABA in PFC, thus reducing the inhibition of amygdala (Lu et al., 2017). Rodent models are usually used to study social behavior disorders because they can simulate human social behavior and provide guiding information for the treatment of human social-emotional disorders. However, current rodent models of PTSD are still unable to simulate real PTSD patients well and can only simulate some features of PTSD. Therefore, more suitable animal models of PTSD still need to be explored.

In addition, several other studies have linked the GABAergic system to PTSD. Spivak et al. (2000) reported significantly elevated levels of dehydroepiandrosterone and dehydroepiandrosterone sulfate in response to GABAARs in the blood of patients with war-related chronic PTSD. These results strongly suggested that the increase in steroids and the inhibition of GABAARs are closely related to the delayed recovery of PTSD patients. A molecular genetic study reported that the polymorphism of the microsatellite sequence at the third end of the GABA β3 subunit is correlated with the incidence of PTSD (Feusner et al., 2001).

#### 2.2.2. Neuroimaging evidence

Currently, intracranial GABA levels can be measured using proton magnetic resonance spectroscopy. Neuroimaging studies of BDZ-GABAA receptors in PTSD are rare. The GABAAR complex includes the GABAergic postsynaptic membrane, GABA receptor and chloride ion channel. When BDZ binds to BDZ receptors on the GABAAR complex, chloride channels open, and GABA binds to GABAAR to increase the frequency of chloride channel opening, which promotes chloride influx in a dual way to enhance central inhibition. Bremner et al. (2000) examined the distribution of benzodiazepine receptor recognition sites (a recognition site on the GABAAR complex) in the PFC of PTSD patients and observed that the density was significantly lower than that of normal controls. It has been suggested that changes in the function of GABAAR in this region may underlie several symptoms of PTSD. However, one study (Randall et al., 1995) showed no increase in anxiety and PTSD symptoms in PTSD patients treated with the GABAAR antagonist flumazenil. Flumazenil can reverse the central sedative effects of BDZ, but the results of this study suggest that PTSD and panic disorder (a type of anxiety disorder) differ in BDZ/GABAA system function. This may indicate the complexity of the pathological mechanism of PTSD. Changes in GABA have also been reported in Magnetic resonance spectroscopy studies on the *in vivo* neurochemistry of PTSD. Rosso et al. (2014) reported a 30% decrease in the insular GABA/creatine ratio in 13 adults with PTSD compared to healthy controls. Male veterans and civilians exhibited lower levels of GABA in the parietal occipital cortex (POC) and the proximal temporal lobe than trauma-exposed controls, while POC-GABA levels were inversely correlated with the severity of sleep symptoms (Meyerhoff et al., 2014). Sheth et al. (2019) observed that the exposure of veterans to trauma might be related to lower GABA/H_2_O and glutamine (Gln)/H_2_O levels in the dorsal ACC (dACC), indicating the interruption of the GABA-Gln-Glu cycle. Moreover, changes in Glu/GABA in the dACC in the PTSD group may indicate an excitation-inhibition (E-I) imbalance (Sheth et al., 2019). The E-I balance can represent the ratio of excitatory and inhibitory inputs to a neuron, and the E-I balance of a single neuron is viewed as a combination of two reaction forces projected on the neuron: excitatory and inhibitory. That is, if the excitatory input level increases, the firing rate of the neuron will increase, and if the inhibitory input level increases, the firing rate of the neuron will decrease accordingly. Furthermore, reduced GABA/H_2_O in the ACC was associated with poor sleep quality in the PTSD group (Meyerhoff et al., 2014). Treatments that restore the GABAergic balance may be particularly effective in reducing sleep symptoms of PTSD. Reduction in GABAergic tone may also disturb the E/I balance, which disrupts not only regional neural activity but also functional connections in large-scale brain networks (Gu et al., 2019). These studies collectively suggest that reduced GABAergic activity may underlie the symptoms of PTSD.

#### 2.2.3. Pharmacological evidence

Pharmacological studies also support the involvement of the GABAergic system in PTSD. Potential drugs targeting the GABAergic system can be seen in Table 1.

**TABLE 1:** Potential drugs targeting GABAergic system.

Category	Medicine	Associated studies
Neurosteroids	Allopregnanolone	Almeida et al., 2021; Chen et al., 2021
Benzodiazepines	Flumazenil	Randall et al., 1995
	Alprazolam	Matar et al., 2009
	Clonazepam	Sutherland and Davidson, 1994; Cates et al., 2004
	Midazolam	Gilbert et al., 2020
GABA re-uptake inhibitor	Tiagabine	Connor et al., 2006; Davidson et al., 2007
GABA-A receptor agonist	Eszopiclone	Pollack et al., 2008
GABA-mimetic	Phenibut	Lapin, 2001
	Piracetam	Uniyal et al., 2019

Neurosteroids, including 5-dihydro-progesterone (5-DHP), allopregnanolone (ALLO), and their stereoisomers, such as progesterone, are synthesized directly by brain neurons (glutamatergic and GABAergic long-projection neurons) in the CNS (Baulieu and Robel, 1990; Agís-Balboa et al., 2006, 2007). Neurosteroids not only act on classical steroid hormone receptors (which regulate gene expression and have long-term effects) but also rapidly regulate neuronal excitability by binding membrane receptors to ion channels. They effectively modulate GABAARs (Belelli and Lambert, 2005). Neurosteroid levels and the expression of GABAARs are affected by normal physiological changes, such as pregnancy and the ovarian cycle (Concas et al., 1998; Maguire et al., 2005), as well as by pathological conditions caused by delayed or traumatic stress, such as anxiety, depression, and PTSD (Romeo et al., 1998; Uzunova et al., 1998; Rasmusson et al., 2006). In a physiological state, the mRNA expression level of the GABAAR γ2 L subunit in the cerebral cortex and hippocampus decreased during pregnancy and recovered to the control level 2 days after delivery. Subchronic administration of finasteride (a 5α-reductase inhibitor) in pregnant rats reduced ALLO in the brain to a greater extent than in plasma and prevented the observed decline in γ2S mRNA during pregnancy (Belelli and Lambert, 2005). Pollack et al. (2008) demonstrated that periodic changes in the specific GABAAR subunit during the estrous cycle in mice lead to periodic changes in the tonic inhibition of hippocampal neurons. In late diabetes (the high progesterone phase), tonic inhibition was increased by increased expression of deltaGABA(A)Rs, and decreased neuronal excitability was reflected in decreased susceptibility to epilepsy and anxiety. Elimination of the circulation of deltaGABA(A)Rs by antisense RNA treatment or gene knockout may prevent decreased excitability in diabetes. In a pathological state, Szabó et al. (2014) found that low levels of ALLO in the cerebrospinal fluid of premenopausal women with PTSD may lead to an imbalance between inhibitory and excitatory neurotransmitters, leading to PTSD re-experience and increased depressive symptoms. Romeo et al. (1998) found that GABA receptor positive modulators 3α,5α-tetrahydrogestrone (3α,5α-THP) and 3α,5β-THP concentrations decreased significantly during depression, while 3β,5α-THP levels increased. This imbalance of neuroactive steroids can be corrected with different antidepressant medications. Moreover, ALLO (3α5α and 3α5β isomers) has a high affinity for GABAAR and promotes the action of GABA at these receptors. In one study (Kamprath et al., 2006), SSRIs treatment induced a normal increase in ALLO levels in the cerebrospinal fluid of depressed patients. Normalization of ALLO levels in the cerebrospinal fluid of depressed patients appears to be sufficient to mediate fluoxetine or fluvoxamine to modulate the antianxiety effects of positive allosteric modulation of GABAAR. Progesterone or its neuroactive metabolite tetrahydrogestrinone can produce anti-anxiety, sedation, anesthesia, analgesic, and anticonvulsant effects in rodents and humans by effectively increasing the flux of Cl^–^ by binding GABA to GABAARs (Belelli et al., 2009). Allopregnanolone regulates neuroendocrine changes induced by the stress response, especially in combination with the GABAAR-mediated feedback mechanism that regulates the HPA axis (Almeida et al., 2021). Studies have demonstrated that low levels of allopregnanolone can improve PTSD symptoms by regulating the activity of GABAARs (Schüle et al., 2014).

Generally, BDZ is widely used to treat anxiety disorders, including PTSD, because of its quick-relief properties. In animal studies, BDZ regulates the function of GABAARs and inhibits the “startle” response caused by the threat from predators (Adamec et al., 2006). The role of GABA in PTSD is also supported by studies on treatment using other GABAergic compounds, such as the selective GABA re-uptake inhibitor tiagabine (Taylor, 2003; Connor et al., 2006). In addition, pharmacological studies have found that the combination of piracetam (a GABA derivative drug) and risperidone can enhance the therapeutic of PTSD (Uniyal et al., 2019). Insomnia is among the main features of PTSD, and pharmacological drugs that regulate GABA effectively alleviate insomnia (Randall et al., 1995; Krystal et al., 2014). Although BDZ effectively reduces anxiety in PTSD patients, it do not affect the core symptoms of the disorder, such as intrusive thinking, numbness, and hyperarousal (Gelpin et al., 1996; Viola et al., 1997; Davidson, 2004). This suggests that drugs targeting GABAA receptors may not be effective in the treatment of PTSD, so future studies could explore drugs targeting other targets of the GABAergic system, such as GABA reuptake inhibitors.

### 2.3. How does the GABAergic system change in PTSD?

#### 2.3.1. HPA axis

Stress responses mediated by the HPA axis are most closely related to psychiatric disorders, such as PTSD and depression (Almeida et al., 2021). There are intricate afferent and efferent connections between the hypothalamus and other brain regions of CNS. Neuroendocrine cells in the hypophysiotrophic area of the hypothalamus have endocrine functions and can produce peptide neurohormones to be transported to the adenohypophysis to regulate the secretion of corresponding hormones (Clarke, 2015). Activation of the HPA axis originates from the release of corticotropin-releasing hormone (CRH) from the paraventricular nucleus (PVN) of the hypothalamus, then CRH induces the release of adrenocorticotropic hormone (ACTH), which stimulates the adrenal gland to release glucocorticoid (de Kloet et al., 2005). Glucocorticoid inhibits the release of CRH in the hypothalamus by activating MR and GR with negative feedback (de Kloet et al., 2005). During acute stress, the HPA axis is activated, which plays an important role in mobilizing energy and maintaining homeostasis. However, exposure to a single but extremely intense traumatic event, combined with chronic stress conditions, can lead to anxiety spectrum disorders or induce PTSD. Patients with PTSD showed abnormal regulation of the HPA axis, such as altered cortisol levels or failure to suppress cortisol release during dexamethasone suppression tests (Almeida et al., 2021). Preclinical and clinical studies have demonstrated that continuous activation of the HPA axis leads to elevated levels of corticosterone, which is strongly associated with stress-related psychiatric disorders (Ouellet-Morin et al., 2011; Rasmusson and Pineles, 2018). In addition, the HPA axis and GABAergic system have mutual regulatory effects.

First, changes in GABA signaling cause HPA axis dysfunction in fear memory. GABAergic connects the brain regions involved in the HPA axis and the PVN, as well as its surrounding regions, including the subventricular area, the anterior hypothalamic area, the dorsomedial nucleus of the hypothalamus, the medial preoptic area, the lateral hypothalamic area, and some subnuclei of the bed nucleus of the stria terminalis. Thus, CRH neurons receive several GABAergic inputs (Cullinan et al., 2008). The fear response is regulated by GABAergic signaling through the HPA axis, mainly through the activation of GABAARs (Brickley and Mody, 2012). Previous studies have demonstrated that CRF plays a part in fear extinction learning. Ouellet-Morin et al. (2011) reported that the absence of either GABAAα1 or NMDAR1 gene expression in CRFergic neurons results in significant and long-term deficiency of fear extinction rather than acquisition or retention.

Besides, the HPA axis regulates stress by regulating GABAergic signaling (Mikkelsen et al., 2008). Studies have reported that the injection of metyrapone, an inhibitor of corticosterone synthesis, can prevent the enhancement of intrinsic excitability in BLA→vHPC pyramidal neurons caused by foot shock. Moreover, the overexpression of GR and MR in amygdala neurons provides the basis for corticosterone regulation of BLA neurons (Kohda et al., 2007; Deppermann et al., 2014). Furthermore, changes in the intrinsic excitability of neurons are regulated by several factors, among which the weakening of inhibitory signals by the GABAAR antagonist bicuculline is an important factor (Song et al., 2017). Long-term corticosterone feeding has been observed to decrease GABAAR-mediated tonic inhibition in amygdala neurons. Moreover, corticosterone can alter the expression and function of GABAAR and drive the GABAAR-mediated chloride current (Liu et al., 2017). Liu et al. (2017) found that the recruitment of GABAA(δ)R by researchers through pharmacological and physiological methods leads to the weakening of GABAergic transmission in LA projection neurons, which indicates the disinhibition of GABAA(δ)R. Activation of GABAA(δ)R reduces the input resistance of local interneurons and inhibits the activation of local interneurons. Deletion of the GABAA(δ)R gene weakened its inhibitory effect on LA interneurons. Therefore, corticosterone may enhance the intrinsic excitability of BLA→vHPC pyramidal neurons by reducing their inhibitory signals. In addition, studies have found that (Verkuyl and Joëls, 2003), parvocellular neurons in the PVN receive hormonal inputs mediated by corticosterone as well as GABAergic inhibitory projection. It has been proven that increases in glucocorticoid levels due to stress can inhibit GABAergic tone on parvocellular hypothalamic neurons (Verkuyl et al., 2005). Interestingly, some current studies (Hartmann et al., 2017) suggest that the regulation of fear and stress-related behaviors by corticosterone and glucocorticoids in the forebrain is mediated more by glutamatergic neurons than by GABA. Therefore, there may be two different explanations for the enhancement of neuronal excitability caused by the HPA axis in different brain regions, and more studies are needed to show which mechanism plays a dominant role in other brain regions in the future.

#### 2.3.2. Endocannabinoid system

The endocannabinoid system (ECS) plays an important role in regulating behaviors such as fear and anxiety (Lafenêtre et al., 2007; Ruehle et al., 2012; Gunduz-Cinar et al., 2013; McLaughlin et al., 2014). Moreover, the abnormal function of this system can cause a series of neuropsychiatric disorders, such as depression and anxiety (Hillard et al., 2012; Mechoulam and Parker, 2013). Studies in the past decade have reported the relationship between The ECS and traumatic stress disorder. Piomelli (2003) and Neumeister et al. (2013) found abnormal levels of endocannabinoids in the blood and their receptors in PTSD patients, suggesting possible changes in the function of the ECS in PTSD patients.

Endocannabinoids mainly include N-arachidonoylethanolamine (AEA) and 2-arachidonoylglycerol (2-AG), which are mainly synthesized at the postsynaptic and serve as retrograde first messengers. It regulates the release of neurotransmitters by activating cannabinoid receptors located in the presynaptic membrane (Piomelli, 2003). Endocannabinoids primarily regulate the transport of GABA and Glu neurotransmitters. In addition, they also regulate the release of acetylcholine, biogenic amines noradrenaline and serotonin, and the neuropeptide CCK-8 (Piomelli, 2003).

There are two main types of cannabinoid receptors in the human body, the CB1 receptor and CB2 receptor, both of which are G-protein-coupled receptors that can activate intracellular signal transduction pathways and regulate the release of neurotransmitters and synaptic function (Adolphs et al., 2002). Animal studies have observed that the knockdown of CB1 receptors or the inhibition of CB1 receptor signaling with drugs can cause anxiety symptoms (Haller et al., 2002, 2004). The CB1 receptor-knockout mice could not eliminate fear memory after fear extinction training, indicating that the CB1 receptor plays an important role in the process of fear extinction (Marsicano et al., 2002; Cannich et al., 2004; Dubreucq et al., 2012). The agonists of CB1 such as nabilone and △9-THC can relieve some of the clinical symptoms of PTSD, such as over-stress, forgetfulness, nightmares, and pain (Bremner et al., 1996; Fraser, 2009; Passie et al., 2012). These findings suggest that the CB1 receptor is involved in regulating the transmission of neural information in the PTSD brain and plays an important role in the pathogenesis of PTSD.

Recent studies have reported that endocannabinoids regulate the processing of fear memory (Marsicano et al., 2002; Barad et al., 2006; Mahan and Ressler, 2012; Drumond et al., 2017). They are involved in the acquisition, consolidation, retrieval, and extinction of fear memories, as well as in the regulation of emotional states (Ruehle et al., 2012; Trezza and Campolongo, 2013; Xu and Südhof, 2013). In the hippocampus and amygdala, the activation of GABAergic presynaptic CB1 receptors decreases the release of GABA transmitters, thereby modulating the processing of fear memories (Morales et al., 2004; Szabó et al., 2014). The CB1 receptors are located in the presynaptic membranes of glutamatergic and GABAergic neurons, and their activation inhibits the release of Glu (Kamprath et al., 2006; Hoffman et al., 2010) and GABA (Laaris et al., 2010). Several researchers have suggested that there exists a dynamic balance system that is composed of Glu and GABA in the brain, which jointly maintains a balance between the inhibition and excitation of nerve cells. Once this dynamic balance is broken, anxiety, depression, and other emotional-related behaviors may occur (Martin et al., 2002; Haller et al., 2004), and the ECB system is an important part of the regulation of this balance. Studies have demonstrated that activation of the CB1 receptor in the dorsolateral periaqueductal gray can induce anxiety-like effects in the elevated plus maze, while the CB1 receptor antagonist AM251 prevents anxiety-like behaviors resulting from the Vogel conflict test (Lisboa et al., 2008). The activation of CB1 receptors on GABAergic neurons leads to the reduced release of GABA transmitters, resulting in anxiety-like responses (Roohbakhsh et al., 2009).

#### 2.3.3. Imbalance between excitatory (Glu) and inhibitory (GABA) transmitters

The main inhibitory neurotransmitter in the brain is GABA, acting along with Glu to control the balance of excitation and inhibition in several brain circuits (Petroff, 2002). The neurotoxicity of Glu not only leads to the apoptosis and necrosis of brain neurons in patients with ischemic cerebrovascular disease (Sun et al., 2015) but also affects synaptic plasticity in the brains of patients with PTSD (Stachowicz, 2018). The GABAergic system is an important inhibitory system in the CNS that can protect the normal regulatory function of the nervous system by inhibiting the hyper-excitatory effect of Glu. During the metabolic switch between Glu and GABA, two pathways eliminate excess Glu in the synaptic cleft or mitigate its neurotoxicity (Gao et al., 2014).

The production of GABA from Glu promotes the release of more Glu, which is mediated by glutamic acid decarboxylase (GAD). The GAD is the key rate-limiting enzyme in GABA synthesis. There are two kinds of isoenzymes of GAD, GAD65 and GAD67. Abnormal function of GAD65/67 can also lead to decreased GABA levels. Generally, GAD65 is primarily responsible for the synthesis of GABA that is released at synapses, while GAD67 is the 67 kDa isoform of GAD (GABA synthase), which is responsible for the synthesis of GABA in the metabolic pool of neurons. The distribution of GAD is consistent with that of GABAergic neurons, which makes GAD an excellent marker enzyme for GABAergic neurons, with the functional status of GABAergic neurons in the CNS being often reflected by measuring the activity of GAD in brain tissue. It was reported that chronic adverse stimulation further decreased the expression level of GAD67 and the number of GAD67-positive neurons, suggesting that stress impaired the conversion of Glu to GABA by reducing the level of GAD, thereby limiting GABA synthesis (Fogaça and Duman, 2019). The levels of GAD67 in the hippocampus, amygdala, and PFC of PTSD rats were decreased, suggesting that the reduction in GABA content could weaken the central inhibitory function and disrupt the balance between GABA and Glu. The results of autopsies of patients with major depressive disorder showed significant reductions in the activity of GAD65 in the PFC brain region (Karolewicz et al., 2010), as well as the density of GAD65/67 in the PVN of the hypothalamus (Gao et al., 2013). Similarly, the expression of GAD67 was downregulated in the PFC (Pochwat et al., 2016) in both mouse and rat models of depression. The GAD65 knockout mice showed fear generalization and fear resolution disorders in preclinical trials (Sangha et al., 2009), suggesting an important role for GAD65 in preventing anxiety-like behaviors, fear memory extinction, and resilience to the development of contextual fear generalization.

The GABA transporter (GAT) may play an important role in mental disorders such as anxiety and depression by affecting GABAergic transmission. However, evidence for this hypothesis is scarce, with studies reporting that patients with anxiety disorders exhibit positive clinical responses to the selective GAT-1 inhibitor tiagabine (Schwartz and Nihalani, 2006). In the study of Liu et al. (2007) the researchers used the forced swimming test and the tail suspension test to model depression and anxiety in mice and measured resilience through the open field test, the dark light exploration test, the emergence test and the elevated plus maze (EPM) behavior test. The GAT-1-deficient mice exhibit increased resilience to acute stress in response to behavioral paradigms such as antidepressant- and antianxiety-like activities (Liu et al., 2007; Gong et al., 2015), which substantiates the role of GAT-1 in mood disorders. However, the role of GAT in PTSD needs investigation. The concentration of GABA in the extra-synaptic space is regulated in several ways. In addition to GAT-1, the GABA transporters GAT-2 and betaine/GABA transporter 1 (BGT-1) are located on astrocytes that recycle GABA. Additionally, GABA in astrocytes can enter the intercellular space through the bestrophin channel as a non-vesicular release (Brickley and Mody, 2012), and these regulatory mechanisms maintain the concentration of GABA at a stable level outside synapses. However, there are very few studies linking GABA- astrocytes to PTSD, which gives us an indication that future studies may focus on this aspect.

Psychological stress can cause an imbalance between excitatory Glu and inhibitory GABA neurotransmitters, which can lead to a series of neurochemical changes. First, the increased expression of apoptotic markers in the muscle, thymus, and nervous system is related to stress. Moreover, the survival and apoptosis of neurons are related to the mechanism of the balance between Glu and GABA (Engelbrecht et al., 2010). The glutamate/gamma-aminobutyric acid-glutamine metabolic circuit in the CNS is the main pathway that regulates the metabolism of Glu and GABA, which is important to maintain the dynamic balance between excitatory and inhibitory systems in the brain (Jiang et al., 2022). During the metabolic conversion of Glu to GABA, excess Glu in the synaptic cleft can be eliminated or reduced through the excitatory amino acid transporters (EAATs) and GAD pathways. In other words, when the conversion level of Glu to GABA is insufficient, resulting in too much Glu and too little GABA in the CNS, an imbalance of excitation and inhibition can occur. The elevation or accumulation of Glu can induce apoptosis in sensitive neural cells. When GABA is catalyzed by Glu and directed by GAD, it has positive feedback on its own release. Such imbalance further leads to dysfunctional excitatory spasms. The findings of Fogaça and Duman (2019) suggest that the imbalance between the excitatory (Glu) and inhibitory (GABA) transmitters may promote apoptosis in the hippocampal neurons and play an important role in the pathogenesis of PTSD. However, this finding is inconsistent with the brain imaging results of patients, which may indicate that humans and rodents have different responses to PTSD-like stress. Therefore, the specific reasons for this need further investigation.

## 3. Clinical directions

Several types of drugs, mainly antidepressants, antipsychotics, and anticonvulsants, target PTSD. However, anti-stress drugs are not widely used in PTSD, although they can regulate the GABAergic system, stimulate GABA receptors, inhibit GABA transporters, and restore the function of the GABAergic system in the brain.

As anti-anxiety agents, BDZ is widely used clinically. For instance, BDZ such as diazepam, flurazepam, clorazepate, oxazepam, and triazolam, is also used in the treatment of PTSD symptoms, but prolonged use of these agents can cause dizziness, drowsiness, fatigue, and other adverse effects. In addition, BDZ promotes GABA-mediated neural transmission in the CNS (Mao et al., 1975). Such drugs do not directly excite GABA recognition sites and accelerate the release of GABA but increase the affinity of low-affinity GABAAR or the number of GABAARs through allosteric regulation to enhance the inhibitory function of GABA. Promoting the E-I balance caused by stress produces anti-anxiety and sedative effects. Studies on the GABAAR family reported that the α2 and α3 subunits are closely related to anxiety behaviors under the effect of BDZ (Dias et al., 2005). The α5 subunit is involved in hippocampus-associated associative memory (Rudolph et al., 1999; Rudolph and Möhler, 2004). The γ^2^ subunit is extensively regulated by BDZ in the brain (Günther et al., 1995).

Topiramate is an anticonvulsant, which is also among the supplementary drugs in the clinical use of PTSD (Khan and Liberzon, 2004). Topiramate enhances the function of the GABA receptor and induces the influx of chloride ions. It is also involved in the intervention of PTSD symptoms by regulating and restoring the content of GABA in the brain and maintaining the excitation-inhibitory stability of Glu and GABA in the CNS (Berlant, 2001). In clinical studies on PTSD, it was observed that the rate of relief from PTSD symptoms in the topiramate-treatment group was significantly higher than that in the control group. Topiramate has been proven to relieve the symptoms of recurrent traumatic experiences, nightmares, and other PTSD symptoms, such as high arousal, although these drugs also have significant side effects, including dizziness and lethargy.

Studies have also found that neurosteroids have an anti-anxiety effect by acting on the GABAergic system, as they can directly act on the GABAAR, prolong the opening time and increase the opening frequency of its chloride channel, thus increasing the inhibitory effect of GABA (Lambert et al., 1995).

## 4. Conclusion

The GABAergic system is significant in the regulation of PTSD. After traumatic events, the formation of fear memory and synaptic plasticity are affected by the GABA inhibition system, and the balance in excitation-inhibition homeostasis between neural networks is destroyed, resulting in complex changes in the thoughts, feelings, and behaviors of the subject. Anti-stress drugs that regulate the GABAergic system restore GABA function in the brain by stimulating GABA receptors or inhibiting GABA transporters. Although such drugs can relieve PTSD symptoms, they can also have side effects such as dizziness, drowsiness, and fatigue. Future research is needed to further explore the common mechanisms underlying the core symptoms of PTSD to guide the discovery of ideal targets for the treatment of PTSD. In summary, the consequence of PTSD may be related to the downregulation of GABAergic system. However, although patients with PTSD have been found reduced GABAARs, the GABAergic system has weaker pharmacodynamics at the receptor level.

## Author contributions

JH and FX conceived and designed the study and wrote the draft. LY and LT collected the literature and organized the structure. XW, ZD, and YZ contributed to the supervision. XY and YL used software to do the drawing. KL and WW reviewed and revised the manuscript. All authors contributed to the article and approved the submitted version.

## References

[B1] Adamec R. Strasser K. Blundell J. Burton P. McKay D. W. (2006). Protein synthesis and the mechanisms of lasting change in anxiety induced by severe stress. *Behav. Brain Res.* 167 270–286. 10.1016/j.bbr.2005.09.019 16256211

[B2] Adolphs R. Baron-Cohen S. Tranel D. (2002). Impaired recognition of social emotions following amygdala damage. *J. Cogn. Neurosci.* 14 1264–1274. 10.1162/089892902760807258 12495531

[B3] Agís-Balboa R. C. Pinna G. Pibiri F. Kadriu B. Costa E. Guidotti A. (2007). Down-regulation of neurosteroid biosynthesis in corticolimbic circuits mediates social isolation-induced behavior in mice. *Proc. Natl. Acad. Sci. U.S.A.* 104 18736–18741. 10.1073/pnas.0709419104 18003893 PMC2141846

[B4] Agís-Balboa R. C. Pinna G. Zhubi A. Maloku E. Veldic M. Costa E. (2006). Characterization of brain neurons that express enzymes mediating neurosteroid biosynthesis. *Proc. Natl. Acad. Sci. U.S.A.* 103 14602–14607. 10.1073/pnas.0606544103 16984997 PMC1600006

[B5] Akirav I. Raizel H. Maroun M. (2006). Enhancement of conditioned fear extinction by infusion of the GABA(A) agonist muscimol into the rat prefrontal cortex and amygdala. *Eur. J. Neurosci.* 23 758–764. 10.1111/j.1460-9568.2006.04603.x 16487156

[B6] Almada R. C. Albrechet-Souza L. Brandão M. L. (2013). Further evidence for involvement of the dorsal hippocampus serotonergic and γ-aminobutyric acid (GABA)ergic pathways in the expression of contextual fear conditioning in rats. *J. Psychopharmacol. (Oxford, England)* 27 1160–1168. 10.1177/0269881113482840 23535348

[B7] Almeida F. B. Pinna G. Barros H. M. T. (2021). The role of HPA axis and allopregnanolone on the neurobiology of major depressive disorders and PTSD. *Int. J. Mol. Sci.* 22:5495. 10.3390/ijms22115495 34071053 PMC8197074

[B8] Almeida M. Sousa Filho L. F. Rabello P. M. Santiago B. M. (2020). International classification of diseases - 11th revision: From design to implementation. *Rev. Saude Publica* 54:104. 10.11606/s1518-8787.2020054002120 33175024 PMC7647465

[B9] Anagnostaras S. G. Maren S. Fanselow M. S. (1999). Temporally graded retrograde amnesia of contextual fear after hippocampal damage in rats: Within-subjects examination. *J. Neurosci.* 19 1106–1114. 10.1523/JNEUROSCI.19-03-01106.1999 9920672 PMC6782148

[B10] Anagnostaras S. G. Wood S. C. Shuman T. Cai D. J. Leduc A. D. Zurn K. R. (2010). Automated assessment of pavlovian conditioned freezing and shock reactivity in mice using the video freeze system. *Front. Behav. Neurosci.* 4:158. 10.3389/fnbeh.2010.00158 20953248 PMC2955491

[B11] Ardi Z. Albrecht A. Richter-Levin A. Saha R. Richter-Levin G. (2016). Behavioral profiling as a translational approach in an animal model of posttraumatic stress disorder. *Neurobiol. Dis.* 88 139–147. 10.1016/j.nbd.2016.01.012 26804028

[B12] Aupperle R. L. Melrose A. J. Stein M. B. Paulus M. P. (2012). Executive function and PTSD: Disengaging from trauma. *Neuropharmacology* 62 686–694. 10.1016/j.neuropharm.2011.02.008 21349277 PMC4719148

[B13] Averill L. A. Purohit P. Averill C. L. Boesl M. A. Krystal J. H. Abdallah C. G. (2017). Glutamate dysregulation and glutamatergic therapeutics for PTSD: Evidence from human studies. *Neurosci. Lett.* 649 147–155. 10.1016/j.neulet.2016.11.064 27916636 PMC5482215

[B14] Babaev O. Piletti Chatain C. Krueger-Burg D. (2018). Inhibition in the amygdala anxiety circuitry. *Exp. Mol. Med.* 50 1–16. 10.1038/s12276-018-0063-8 29628509 PMC5938054

[B15] Ball T. M. Knapp S. E. Paulus M. P. Stein M. B. (2017). Brain activation during fear extinction predicts exposure success. *Depress. Anxiety* 34 257–266. 10.1002/da.22583 27921340

[B16] Barad M. Gean P. W. Lutz B. (2006). The role of the amygdala in the extinction of conditioned fear. *Biol. Psychiatry* 60 322–328. 10.1016/j.biopsych.2006.05.029 16919522

[B17] Bast T. Zhang W. N. Feldon J. (2001). The ventral hippocampus and fear conditioning in rats. Different anterograde amnesias of fear after tetrodotoxin inactivation and infusion of the GABA(A) agonist muscimol. *Exp. Brain Res.* 139 39–52. 10.1007/s002210100746 11482842

[B18] Battle D. E. (2013). Diagnostic and statistical manual of mental disorders (DSM). *CoDAS* 25 191–192. 10.1590/s2317-17822013000200017 24413388

[B19] Baulieu E. E. Robel P. (1990). Neurosteroids: A new brain function? *J. Steroid Biochem. Mol. Biol.* 37 395–403. 10.1016/0960-0760(90)90490-c 2257243

[B20] Belelli D. Lambert J. J. (2005). Neurosteroids: Endogenous regulators of the GABA(A) receptor. *Nat. Rev. Neurosci.* 6 565–575. 10.1038/nrn1703 15959466

[B21] Belelli D. Harrison N. L. Maguire J. Macdonald R. L. Walker M. C. Cope D. W. (2009). Extrasynaptic GABAA receptors: Form, pharmacology, and function. *J. Neurosci.* 29 12757–12763. 10.1523/JNEUROSCI.3340-09.2009 19828786 PMC2784229

[B22] Benjet C. Bromet E. Karam E. G. Kessler R. C. McLaughlin K. A. Ruscio A. M. (2016). The epidemiology of traumatic event exposure worldwide: results from the World Mental Health Survey Consortium. *Psychol. Med.* 46 327–343. 10.1017/S0033291715001981 26511595 PMC4869975

[B23] Berlant J. L. (2001). Topiramate in posttraumatic stress disorder: Preliminary clinical observations. *J. Clin. Psychiatry* 62 Suppl 17 60–63. 11495099

[B24] Berlau D. J. McGaugh J. L. (2006). Enhancement of extinction memory consolidation: The role of the noradrenergic and GABAergic systems within the basolateral amygdala. *Neurobiol. Learn. Mem.* 86 123–132. 10.1016/j.nlm.2005.12.008 16458544

[B25] Bhagwagar Z. Wylezinska M. Jezzard P. Evans J. Boorman E. Matthews P. (2008). Low GABA concentrations in occipital cortex and anterior cingulate cortex in medication-free, recovered depressed patients. *Int. J. Neuropsychopharmacol.* 11 255–260. 10.1017/S1461145707007924 17625025

[B26] Bishop J. R. Lee A. M. Mills L. J. Thuras P. D. Eum S. Clancy D. (2021). Corrigendum: Methylation of FKBP5 and SLC6A4 in relation to treatment response to mindfulness based stress reduction for posttraumatic stress disorder. *Front. Psychiatry* 12:642245. 10.3389/fpsyt.2021.642245 33746798 PMC7970629

[B27] Blanchard R. J. Blanchard D. C. (1989). Attack and defense in rodents as ethoexperimental models for the study of emotion. *Prog. Neuro Psychopharmacol. Biol. Psychiatry* 13 Suppl S3–S14. 10.1016/0278-5846(89)90105-x 2694228

[B28] Bouton M. Bolles R. (1980). Conditioned fear assessed by freezing and by the suppression of three different baselines. *Anim. Learn. Behav.* 8 429–434.

[B29] Bremner J. D. Innis R. B. Southwick S. M. Staib L. Zoghbi S. Charney D. S. (2000). Decreased benzodiazepine receptor binding in prefrontal cortex in combat-related posttraumatic stress disorder. *Am. J. Psychiatry* 157 1120–1126. 10.1176/appi.ajp.157.7.1120 10873921

[B30] Bremner J. D. Southwick S. M. Darnell A. Charney D. S. (1996). Chronic PTSD in Vietnam combat veterans: Course of illness and substance abuse. *Am. J. Psychiatry* 153 369–375. 10.1176/ajp.153.3.369 8610824

[B31] Brickley S. G. Mody I. (2012). Extrasynaptic GABA(A) receptors: Their function in the CNS and implications for disease. *Neuron* 73 23–34. 10.1016/j.neuron.2011.12.012 22243744 PMC3399243

[B32] Cannich A. Wotjak C. T. Kamprath K. Hermann H. Lutz B. Marsicano G. (2004). CB1 cannabinoid receptors modulate kinase and phosphatase activity during extinction of conditioned fear in mice. *Learn. Mem. (Cold Spring Harbor, N.Y.)* 11 625–632. 10.1101/lm.77904 15466318 PMC523082

[B33] Castellano C. McGaugh J. L. (1990). Effects of post-training bicuculline and muscimol on retention: Lack of state dependency. *Behav. Neural Biol.* 54 156–164. 10.1016/0163-1047(90)91352-c 2173543

[B34] Catarino A. Küpper C. S. Werner-Seidler A. Dalgleish T. Anderson M. C. (2015). Failing to forget: Inhibitory-control deficits compromise memory suppression in posttraumatic stress disorder. *Psychol. Sci.* 26 604–616. 10.1177/0956797615569889Meiser-Stedman 25847536 PMC4426138

[B35] Cates M. E. Bishop M. H. Davis L. L. Lowe J. S. Woolley T. W. (2004). Clonazepam for treatment of sleep disturbances associated with combat-related posttraumatic stress disorder. *Ann. Pharmacother.* 38 1395–1399. 10.1345/aph.1E043 15252193

[B36] Charney D. S. (2004). Psychobiological mechanisms of resilience and vulnerability: Implications for successful adaptation to extreme stress. *Am. J. Psychiatry* 161 195–216. 10.1176/appi.ajp.161.2.195 14754765

[B37] Chebib M. Johnston G. A. (1999). The ‘ABC’ of GABA receptors: A brief review. *Clin. Exp. Pharmacol. Physiol.* 26 937–940. 10.1046/j.1440-1681.1999.03151.x 10561820

[B38] Chen S. W. Shemyakin A. Wiedenmayer C. P. (2006). The role of the amygdala and olfaction in unconditioned fear in developing rats. *J. Neurosci.* 26 233–240. 10.1523/JNEUROSCI.2890-05.2006 16399692 PMC6674335

[B39] Chen S. Gao L. Li X. Ye Y. (2021). Allopregnanolone in mood disorders: Mechanism and therapeutic development. *Pharmacol. Res.* 169:105682. 10.1016/j.phrs.2021.105682 34019980

[B40] Christianson J. Fernando A. Kazama A. Jovanovic T. Ostroff L. Sangha S. (2012). Inhibition of fear by learned safety signals: A mini-symposium review. *J. Neurosci.* 32 14118–14124. 10.1523/JNEUROSCI.3340-12.2012 23055481 PMC3541026

[B41] Clarke I. J. (2015). Hypothalamus as an endocrine organ. *Compr. Physiol.* 5 217–253. 10.1002/cphy.c140019 25589270

[B42] Concas A. Mostallino M. C. Porcu P. Follesa P. Barbaccia M. L. Trabucchi M. (1998). Role of brain allopregnanolone in the plasticity of gamma-aminobutyric acid type A receptor in rat brain during pregnancy and after delivery. *Proc. Natl. Acad. Sci. U.S.A.* 95 13284–13289. 10.1073/pnas.95.22.13284 9789080 PMC23784

[B43] Connor K. M. Davidson J. R. Weisler R. H. Zhang W. Abraham K. (2006). Tiagabine for posttraumatic stress disorder: Effects of open-label and double-blind discontinuation treatment. *Psychopharmacology* 184 21–25. 10.1007/s00213-005-0265-3 16341846

[B44] Corcoran K. A. Maren S. (2001). Hippocampal inactivation disrupts contextual retrieval of fear memory after extinction. *J. Neurosci.* 21 1720–1726. 10.1523/JNEUROSCI.21-05-01720.2001 11222661 PMC6762930

[B45] Craddock N. Jones L. Jones I. R. Kirov G. Green E. K. Grozeva D. (2010). Strong genetic evidence for a selective influence of GABAA receptors on a component of the bipolar disorder phenotype. *Mol. Psychiatry* 15 146–153. 10.1038/mp.2008.66 19078961 PMC3967096

[B46] Cullinan W. E. Ziegler D. R. Herman J. P. (2008). Functional role of local GABAergic influences on the HPA axis. *Brain Struct. Funct.* 213 63–72. 10.1007/s00429-008-0192-2 18696110

[B47] Davidson J. R. (2004). Use of benzodiazepines in social anxiety disorder, generalized anxiety disorder, and posttraumatic stress disorder. *J. Clin. Psychiatry* 65 Suppl 5 29–33. 15078116

[B48] Davidson J. R. Brady K. Mellman T. A. Stein M. B. Pollack M. H. (2007). The efficacy and tolerability of tiagabine in adult patients with post-traumatic stress disorder. *J. Clin. Psychopharmacol.* 27 85–88. 10.1097/JCP.0b013e31802e5115 17224720

[B49] Davidson R. J. Putnam K. M. Larson C. L. (2000). Dysfunction in the neural circuitry of emotion regulation–A possible prelude to violence. *Science (New York, N.Y.)* 289 591–594. 10.1126/science.289.5479.591 10915615

[B50] Davis M. Walker D. L. Miles L. Grillon C. (2010). Phasic vs sustained fear in rats and humans: Role of the extended amygdala in fear vs anxiety. *Neuropsychopharmacology* 35 105–135. 10.1038/npp.2009.109 19693004 PMC2795099

[B51] de Kloet E. R. Joëls M. Holsboer F. (2005). Stress and the brain: From adaptation to disease. *Nat. Rev. Neurosci.* 6 463–475. 10.1038/nrn1683 15891777

[B52] Deppermann S. Storchak H. Fallgatter A. J. Ehlis A. C. (2014). Stress-induced neuroplasticity: (Mal)adaptation to adverse life events in patients with PTSD–a critical overview. *Neuroscience* 283 166–177. 10.1016/j.neuroscience.2014.08.037 25193848

[B53] Dias R. Sheppard W. F. Fradley R. L. Garrett E. M. Stanley J. L. Tye S. J. (2005). Evidence for a significant role of alpha 3-containing GABAA receptors in mediating the anxiolytic effects of benzodiazepines. *J. Neurosci.* 25 10682–10688. 10.1523/JNEUROSCI.1166-05.2005 16291941 PMC6725841

[B54] Disner S. G. Marquardt C. A. Mueller B. A. Burton P. C. Sponheim S. R. (2018). Spontaneous neural activity differences in posttraumatic stress disorder: A quantitative resting-state meta-analysis and fMRI validation. *Hum. Brain Mapp.* 39 837–850. 10.1002/hbm.23886 29143411 PMC6866285

[B55] Dolfen N. Veldman M. P. Gann M. A. von Leupoldt A. Puts N. A. J. Edden R. A. E. (2021). A role for GABA in the modulation of striatal and hippocampal systems under stress. *Commun. Biol.* 4:1033. 10.1038/s42003-021-02535-x 34475515 PMC8413374

[B56] Domschke K. Zwanzger P. (2008). GABAergic and endocannabinoid dysfunction in anxiety – Future therapeutic targets? *Curr. Pharm. Design* 14 3508–3517. 10.2174/138161208786848784 19075727

[B57] Domschke K. Tidow N. Schwarte K. Deckert J. Lesch K. Arolt V. (2014). Serotonin transporter gene hypomethylation predicts impaired antidepressant treatment response. *Int. J. Neuropsychopharmacol.* 17 1167–1176. 10.1017/S146114571400039X 24679990

[B58] Drugan R. C. Basile A. S. Crawley J. N. Paul S. M. Skolnick P. (1986). Inescapable shock reduces [3H]Ro 5-4864 binding to “peripheral-type” benzodiazepine receptors in the rat. *Pharmacol. Biochem. Behav.* 24 1673–1677. 10.1016/0091-3057(86)90504-6 3016760

[B59] Drumond A. Madeira N. Fonseca R. (2017). Endocannabinoid signaling and memory dynamics: A synaptic perspective. *Neurobiol. Learn. Mem.* 138 62–77. 10.1016/j.nlm.2016.07.031 27481224

[B60] Dubreucq S. Matias I. Cardinal P. Häring M. Lutz B. Marsicano G. (2012). Genetic dissection of the role of cannabinoid type-1 receptors in the emotional consequences of repeated social stress in mice. *Neuropsychopharmacology* 37 1885–1900. 10.1038/npp.2012.36 22434220 PMC3376321

[B61] Elms L. Shannon S. Hughes S. Lewis N. (2019). Cannabidiol in the treatment of post-traumatic stress disorder: A case series. *J. Altern. Complement. Med.* 25 392–397. 30543451 10.1089/acm.2018.0437 PMC6482919

[B62] Engelbrecht A. M. Smith C. Neethling I. Thomas M. Ellis B. Mattheyse M. (2010). Daily brief restraint stress alters signaling pathways and induces atrophy and apoptosis in rat skeletal muscle. *Stress (Amsterdam, Netherlands)* 13 132–141. 10.3109/10253890903089834 19929313

[B63] Evans J. Sun Y. McGregor A. Connor B. (2012). Allopregnanolone regulates neurogenesis and depressive/anxiety-like behaviour in a social isolation rodent model of chronic stress. *Neuropharmacology* 63 1315–1326. 10.1016/j.neuropharm.2012.08.012 22939998

[B64] Fanselow M. S. Dong H. W. (2010). Are the dorsal and ventral hippocampus functionally distinct structures? *Neuron* 65 7–19. 10.1016/j.neuron.2009.11.031 20152109 PMC2822727

[B65] Fendt M. Fanselow M. S. (1999). The neuroanatomical and neurochemical basis of conditioned fear. *Neurosci. Biobehav. Rev.* 23 743–760. 10.1016/s0149-7634(99)00016-0 10392663

[B66] Ferrara N. C. Jarome T. J. Cullen P. K. Orsi S. A. Kwapis J. L. Trask S. (2019). GluR2 endocytosis-dependent protein degradation in the amygdala mediates memory updating. *Sci. Rep.* 9:5180. 10.1038/s41598-019-41526-1 30914678 PMC6435726

[B67] Feusner J. Ritchie T. Lawford B. Young R. M. Kann B. Noble E. P. (2001). GABA(A) receptor beta 3 subunit gene and psychiatric morbidity in a post-traumatic stress disorder population. *Psychiatry Res.* 104 109–117. 10.1016/s0165-1781(01)00296-7 11711165

[B68] Fogaça M. V. Duman R. S. (2019). Cortical GABAergic dysfunction in stress and depression: New insights for therapeutic interventions. *Front. cell. Neurosci.* 13:87. 10.3389/fncel.2019.00087 30914923 PMC6422907

[B69] Fraser G. A. (2009). The use of a synthetic cannabinoid in the management of treatment-resistant nightmares in posttraumatic stress disorder (PTSD). *CNS Neurosci. Ther.* 15 84–88. 10.1111/j.1755-5949.2008.00071.x 19228182 PMC6494011

[B70] Gao J. Wang H. Liu Y. Li Y. Y. Chen C. Liu L. M. (2014). Glutamate and GABA imbalance promotes neuronal apoptosis in hippocampus after stress. *Med. Sci. Monit.* 20 499–512. 10.12659/MSM.890589 24675061 PMC3976216

[B71] Gao S. F. Klomp A. Wu J. L. Swaab D. F. Bao A. M. (2013). Reduced GAD(65/67) immunoreactivity in the hypothalamic paraventricular nucleus in depression: A postmortem study. *J. Affect. Disord.* 149 422–425. 10.1016/j.jad.2012.12.003 23312397

[B72] Gelpin E. Bonne O. Peri T. Brandes D. Shalev A. Y. (1996). Treatment of recent trauma survivors with benzodiazepines: A prospective study. *J. Clin. Psychiatry* 57 390–394. 9746445

[B73] Geracioti T. D. Jr. Baker D. G. Kasckow J. W. Strawn J. R. Jeffrey Mulchahey J. Dashevsky B. A. (2008). Effects of trauma-related audiovisual stimulation on cerebrospinal fluid norepinephrine and corticotropin-releasing hormone concentrations in post-traumatic stress disorder. *Psychoneuroendocrinology* 33 416–424. 10.1016/j.psyneuen.2007.12.012 18295412

[B74] Geuze E. van Berckel B. N. Lammertsma A. A. Boellaard R. de Kloet C. S. Vermetten E. (2008). Reduced GABAA benzodiazepine receptor binding in veterans with post-traumatic stress disorder. *Mol. Psychiatry* 13 74–83, 3. 10.1038/sj.mp.4002054 17667960

[B75] Ghosal S. Hare B. Duman R. S. (2017). Prefrontal cortex GABAergic deficits and circuit dysfunction in the pathophysiology and treatment of chronic stress and depression. *Curr. Opin. Behav. Sci.* 14 1–8. 10.1016/j.cobeha.2016.09.012 27812532 PMC5086803

[B76] Gilbert M. Dinh La A. Romulo Delapaz N. Kenneth Hor W. Fan P. Qi X. (2020). An emulation of randomized trials of administrating benzodiazepines in PTSD patients for outcomes of suicide-related events. *J. Clin. Med.* 9:3492. 10.3390/jcm9113492 33138006 PMC7694098

[B77] Gilmartin M. R. Kwapis J. L. Helmstetter F. J. (2012). Trace and contextual fear conditioning are impaired following unilateral microinjection of muscimol in the ventral hippocampus or amygdala, but not the medial prefrontal cortex. *Neurobiol. Learn. Mem.* 97 452–464. 10.1016/j.nlm.2012.03.009 22469748 PMC3358523

[B78] Gong X. Shao Y. Li B. Chen L. Wang C. Chen Y. (2015). γ-aminobutyric acid transporter-1 is involved in anxiety-like behaviors and cognitive function in knockout mice. *Exp. Ther. Med.* 10 653–658. 10.3892/etm.2015.2577 26622370 PMC4509144

[B79] Gu H. Hu Y. Chen X. He Y. Yang Y. (2019). Regional excitation-inhibition balance predicts default-mode network deactivation via functional connectivity. *NeuroImage* 185 388–397. 10.1016/j.neuroimage.2018.10.055 30359729 PMC8284909

[B80] Gunduz-Cinar O. Hill M. N. McEwen B. S. Holmes A. (2013). Amygdala FAAH and anandamide: Mediating protection and recovery from stress. *Trends Pharmacol. Sci.* 34 637–644. 10.1016/j.tips.2013.08.008 24325918 PMC4169112

[B81] Günther U. Benson J. Benke D. Fritschy J. M. Reyes G. Knoflach F. (1995). Benzodiazepine-insensitive mice generated by targeted disruption of the gamma 2 subunit gene of gamma-aminobutyric acid type A receptors. *Proc. Natl. Acad. Sci. U.S.A.* 92 7749–7753. 10.1073/pnas.92.17.7749 7644489 PMC41223

[B82] Hageman I. Andersen H. S. Jørgensen M. B. (2001). Post-traumatic stress disorder: A review of psychobiology and pharmacotherapy. *Acta Psychiatr. Scand.* 104 411–422. 10.1034/j.1600-0447.2001.00237.x 11782234

[B83] Haller J. Bakos N. Szirmay M. Ledent C. Freund T. F. (2002). The effects of genetic and pharmacological blockade of the CB1 cannabinoid receptor on anxiety. *Eur. J. Neurosci.* 16 1395–1398. 10.1046/j.1460-9568.2002.02192.x 12405999

[B84] Haller J. Varga B. Ledent C. Freund T. F. (2004). CB1 cannabinoid receptors mediate anxiolytic effects: Convergent genetic and pharmacological evidence with CB1-specific agents. *Behav. Pharmacol.* 15 299–304. 10.1097/01.fbp.0000135704.56422.40 15252281

[B85] Hartmann J. Dedic N. Pöhlmann M. L. Häusl A. Karst H. Engelhardt C. (2017). Forebrain glutamatergic, but not GABAergic, neurons mediate anxiogenic effects of the glucocorticoid receptor. *Mol. Psychiatry* 22 466–475. 10.1038/mp.2016.87 27240530

[B86] Harvey B. H. Oosthuizen F. Brand L. Wegener G. Stein D. J. (2004). Stress-restress evokes sustained iNOS activity and altered GABA levels and NMDA receptors in rat hippocampus. *Psychopharmacology* 175 494–502. 10.1007/s00213-004-1836-4 15138761

[B87] Hayes J. P. Hayes S. M. Mikedis A. M. (2012). Quantitative meta-analysis of neural activity in posttraumatic stress disorder. *Biol. Mood Anxiety Disord.* 2:9. 10.1186/2045-5380-2-9 22738125 PMC3430553

[B88] Herbst M. R. Twining R. C. Gilmartin M. R. (2022). Ventral hippocampal shock encoding modulates the expression of trace cued fear. *Neurobiol. Learn. Mem.* 190:107610. 10.1016/j.nlm.2022.107610 35302040

[B89] Hill M. N. Campolongo P. Yehuda R. Patel S. (2018). Integrating endocannabinoid signaling and cannabinoids into the biology and treatment of posttraumatic stress disorder. *Neuropsychopharmacology* 43 80–102. 10.1038/npp.2017.162 28745306 PMC5719095

[B90] Hillard C. J. Weinlander K. M. Stuhr K. L. (2012). Contributions of endocannabinoid signaling to psychiatric disorders in humans: Genetic and biochemical evidence. *Neuroscience* 204 207–229. 10.1016/j.neuroscience.2011.11.020 22123166 PMC3288440

[B91] Hoffman A. F. Laaris N. Kawamura M. Masino S. A. Lupica C. R. (2010). Control of cannabinoid CB1 receptor function on glutamate axon terminals by endogenous adenosine acting at A1 receptors. *J. Neurosci.* 30 545–555. 10.1523/JNEUROSCI.4920-09.2010 20071517 PMC2855550

[B92] Ishikawa A. Nakamura S. (2006). Ventral hippocampal neurons project axons simultaneously to the medial prefrontal cortex and amygdala in the rat. *J. Neurophysiol.* 96 2134–2138. 10.1152/jn.00069.2006 16837666

[B93] Izquierdo I. Furini C. R. Myskiw J. C. (2016). Fear memory. *Physiol. Rev.* 96 695–750. 10.1152/physrev.00018.2015 26983799

[B94] Jacob T. C. Moss S. J. Jurd R. (2008). GABA(A) receptor trafficking and its role in the dynamic modulation of neuronal inhibition. *Nat. Rev. Neurosci.* 9 331–343. 10.1038/nrn2370 18382465 PMC2709246

[B95] Jiang C. Wang H. Qi J. Li J. He Q. Wang C. (2022). Antidepressant effects of cherry leaf decoction on a chronic unpredictable mild stress rat model based on the Glu/GABA-Gln metabolic loop. *Metab. Brain Dis.* 37 2883–2901. 10.1007/s11011-022-01081-7 36181653

[B96] Kamprath K. Marsicano G. Tang J. Monory K. Bisogno T. Di Marzo V. (2006). Cannabinoid CB1 receptor mediates fear extinction via habituation-like processes. *J. Neurosci.* 26 6677–6686. 10.1523/JNEUROSCI.0153-06.2006 16793875 PMC6673838

[B97] Karam E. G. Friedman M. J. Hill E. D. Kessler R. C. McLaughlin K. A. Petukhova M. (2014). Cumulative traumas and risk thresholds: 12-month PTSD in the World Mental Health (WMH) surveys. *Depress. Anxiety* 31 130–142. 10.1002/da.22169 23983056 PMC4085043

[B98] Karolewicz B. Maciag D. O’Dwyer G. Stockmeier C. A. Feyissa A. M. Rajkowska G. (2010). Reduced level of glutamic acid decarboxylase-67 kDa in the prefrontal cortex in major depression. *Int. J. Neuropsychopharmacol.* 13 411–420. 10.1017/S1461145709990587 20236554 PMC2857696

[B99] Kato M. Serretti A. (2010). Review and meta-analysis of antidepressant pharmacogenetic fifindings in major depressive disorder. *Mol. Psychiatry* 15 473–500. 10.1038/mp.2008.116 18982004

[B100] Khan S. Liberzon I. (2004). Topiramate attenuates exaggerated acoustic startle in an animal model of PTSD. *Psychopharmacology* 172 225–229. 10.1007/s00213-003-1634-4 14586539

[B101] Klempan T. A. Sequeira A. Canetti L. Lalovic A. Ernst C. ffrench-Mullen J. (2009). Altered expression of genes involved in ATP biosynthesis and GABAergic neurotransmission in the ventral prefrontal cortex of suicides with and without major depression. *Mol. Psychiatry* 14 175–189. 10.1038/sj.mp.4002110 17938633

[B102] Koch M. Schnitzler H. U. (1997). The acoustic startle response in rats–circuits mediating evocation, inhibition and potentiation. *Behav. Brain Res.* 89 35–49. 10.1016/s0166-4328(97)02296-1 9475613

[B103] Koenigs M. Grafman J. (2009). Posttraumatic stress disorder: The role of medial prefrontal cortex and amygdala. *Neuroscientist* 15 540–548. 19359671 10.1177/1073858409333072 PMC2771687

[B104] Kohda K. Harada K. Kato K. Hoshino A. Motohashi J. Yamaji T. (2007). Glucocorticoid receptor activation is involved in producing abnormal phenotypes of single-prolonged stress rats: A putative post-traumatic stress disorder model. *Neuroscience* 148 22–33. 10.1016/j.neuroscience.2007.05.041 17644267

[B105] Krystal A. D. Zhang W. Davidson J. R. Connor K. M. (2014). The sleep effects of tiagabine on the first night of treatment predict post-traumatic stress disorder response at three weeks. *J. Psychopharmacol. (Oxford, England)* 28 457–465. 10.1177/0269881113509903 24288237

[B106] Krystal J. H. Sanacora G. Blumberg H. Anand A. Charney D. S. Marek G. (2002). Glutamate and GABA systems as targets for novel antidepressant and mood-stabilizing treatments. *Mol. Psychiatry* 7 Suppl 1 S71–S80. 10.1038/sj.mp.4001021 11986998

[B107] Kyd R. J. Bilkey D. K. (2005). Hippocampal place cells show increased sensitivity to changes in the local environment following prefrontal cortex lesions. *Cereb. Cortex (New York, N.Y. : 1991)* 15 720–731. 10.1093/cercor/bhh173 15371292

[B108] Laaris N. Good C. H. Lupica C. R. (2010). Delta9-tetrahydrocannabinol is a full agonist at CB1 receptors on GABA neuron axon terminals in the hippocampus. *Neuropharmacology* 59 121–127. 10.1016/j.neuropharm.2010.04.013 20417220 PMC2882293

[B109] Lafenêtre P. Chaouloff F. Marsicano G. (2007). The endocannabinoid system in the processing of anxiety and fear and how CB1 receptors may modulate fear extinction. *Pharmacol. Res.* 56 367–381. 10.1016/j.phrs.2007.09.006 17951068

[B110] Lambert J. J. Belelli D. Hill-Venning C. Peters J. A. (1995). Neurosteroids and GABAA receptor function. *Trends Pharmacol. Sci.* 16 295–303. 10.1016/s0165-6147(00)89058-6 7482994

[B111] Lapin I. (2001). Phenibut (beta-phenyl-GABA): A tranquilizer and nootropic drug. *CNS Drug Rev.* 7 471–481. 10.1111/j.1527-3458.2001.tb00211.x 11830761 PMC6494145

[B112] LeDoux J. E. (2000). Emotion circuits in the brain. *Annu. Rev. Neurosci.* 23 155–184. 10.1146/annurev.neuro.23.1.155 10845062

[B113] LeDoux J. E. Iwata J. Cicchetti P. Reis D. J. (1988). Different projections of the central amygdaloid nucleus mediate autonomic and behavioral correlates of conditioned fear. *J. Neurosci.* 8 2517–2529. 10.1523/JNEUROSCI.08-07-02517.1988 2854842 PMC6569498

[B114] Lehner M. Wisłowska-Stanek A. Skórzewska A. Maciejak P. Szyndler J. Turzyńska D. (2010). Differences in the density of GABA-A receptor alpha-2 subunits and gephyrin in brain structures of rats selected for low and high anxiety in basal and fear-stimulated conditions, in a model of contextual fear conditioning. *Neurobiol. Learn. Mem.* 94 499–508. 10.1016/j.nlm.2010.09.001 20833253

[B115] Levinson A. J. Fitzgerald P. B. Favalli G. Blumberger D. M. Daigle M. Daskalakis Z. J. (2010). Evidence of cortical inhibitory deficits in major depressive disorder. *Biol. Psychiatry* 67 458–464. 10.1016/j.biopsych.2009.09.025 19922906

[B116] Liberzon I. Abelson J. L. (2016). Context processing and the neurobiology of post-traumatic stress disorder. *Neuron* 92 14–30. 10.1016/j.neuron.2016.09.039 27710783 PMC5113735

[B117] Lin H. C. Tseng Y. C. Mao S. C. Chen P. S. Gean P. W. (2011). GABAA receptor endocytosis in the basolateral amygdala is critical to the reinstatement of fear memory measured by fear-potentiated startle. *J. Neurosci.* 31 8851–8861. 10.1523/JNEUROSCI.0979-11.2011 21677169 PMC6622947

[B118] Lippa A. S. Klepner C. A. Yunger L. Sano M. C. Smith W. V. Beer B. (1978). Relationship between benzodiazepine receptors and experimental anxiety in rats. *Pharmacol. Biochem. Behav.* 9 853–856. 10.1016/0091-3057(78)90368-4 34175

[B119] Lisboa S. F. Resstel L. B. Aguiar D. C. Guimarães F. S. (2008). Activation of cannabinoid CB1 receptors in the dorsolateral periaqueductal gray induces anxiolytic effects in rats submitted to the Vogel conflict test. *Eur. J. Pharmacol.* 593 73–78. 10.1016/j.ejphar.2008.07.032 18691568

[B120] Lissek S. van Meurs B. (2015). Learning models of PTSD: Theoretical accounts and psychobiological evidence. *Int. J. Psychophysiol.* 98 (3 Pt 2) 594–605. 10.1016/j.ijpsycho.2014.11.006 25462219 PMC4809259

[B121] Liu G. X. Cai G. Q. Cai Y. Q. Sheng Z. J. Jiang J. Mei Z. (2007). Reduced anxiety and depression-like behaviors in mice lacking GABA transporter subtype 1. *Neuropsychopharmacology* 32 1531–1539. 10.1038/sj.npp.1301281 17164814

[B122] Liu L. Wang L. Cao C. Cao X. Zhu Y. Liu P. (2018). Serotonin transporter 5-HTTLPR genotype is associated with intrusion and avoidance symptoms of DSM-5 posttraumatic stress disorder (PTSD) in Chinese earthquake survivors. *Anxiety Stress Coping* 31 318–327. 10.1080/10615806.2017.1420174 29280387

[B123] Liu Z. P. He Q. H. Pan H. Q. Xu X. B. Chen W. B. He Y. (2017). Delta subunit-containing gamma-aminobutyric acid a receptor disinhibits lateral amygdala and facilitates fear expression in mice. *Biol. Psychiatry* 81 990–1002. 10.1016/j.biopsych.2016.06.022 27591789

[B124] Lu C. Y. Liu X. Jiang H. Pan F. Ho C. S. Ho R. C. (2017). Effects of traumatic stress induced in the juvenile period on the expression of gamma-aminobutyric acid receptor type a subunits in adult rat brain. *Neural Plast.* 2017:5715816. 10.1155/2017/5715816 28352479 PMC5352903

[B125] Lujan R. Ciruela F. (2012). GABAB receptors-associated proteins: Potential drug targets in neurological disorders? *Curr. Drug Targets* 13 129–144. 10.2174/138945012798868425 22023408

[B126] Maddox S. A. Hartmann J. Ross R. A. Ressler K. J. (2019). Deconstructing the gestalt: Mechanisms of fear, threat, and trauma memory encoding. *Neuron* 102 60–74. 10.1016/j.neuron.2019.03.017 30946827 PMC6450587

[B127] Maercker A. (2021). Development of the new CPTSD diagnosis for ICD-11. *Borderline Pers. Disord. Emot. Dysregul.* 8:7. 10.1186/s40479-021-00148-8 33641675 PMC7919312

[B128] Maggio N. Segal M. (2007). Striking variations in corticosteroid modulation of long-term potentiation along the septotemporal axis of the hippocampus. *J. Neurosci.* 27 5757–5765. 10.1523/JNEUROSCI.0155-07.2007 17522319 PMC6672761

[B129] Maguire J. L. Stell B. M. Rafizadeh M. Mody I. (2005). Ovarian cycle-linked changes in GABA(A) receptors mediating tonic inhibition alter seizure susceptibility and anxiety. *Nat. Neurosci.* 8 797–804. 10.1038/nn1469 15895085

[B130] Mahan A. L. Ressler K. J. (2012). Fear conditioning, synaptic plasticity and the amygdala: Implications for posttraumatic stress disorder. *Trends Neurosci.* 35 24–35. 10.1016/j.tins.2011.06.007 21798604 PMC3206195

[B131] Makkar S. R. Zhang S. Q. Cranney J. (2010). Behavioral and neural analysis of GABA in the acquisition, consolidation, reconsolidation, and extinction of fear memory. *Neuropsychopharmacology* 35 1625–1652. 10.1038/npp.2010.53 20410874 PMC3055480

[B132] Mao C. C. Guidotti A. Costa E. (1975). Evidence for an involvement of GABA in the mediation of the cerebellar cGMP decrease and the anticonvulsant action diazepam. *Naunyn Schmiedebergs Arch. Pharmacol.* 289 369–378. 10.1007/BF00508411 240134

[B133] Maren S. Quirk G. J. (2004). Neuronal signalling of fear memory. *Nat. Rev. Neurosci.* 5 844–852. 10.1038/nrn1535 15496862

[B134] Maren S. Yap S. A. Goosens K. A. (2001). The amygdala is essential for the development of neuronal plasticity in the medial geniculate nucleus during auditory fear conditioning in rats. *J. Neurosci.* 21:RC135. 10.1523/JNEUROSCI.21-06-j0001.2001 11245704 PMC6762621

[B135] Marquis J. P. Killcross S. Haddon J. E. (2007). Inactivation of the prelimbic, but not infralimbic, prefrontal cortex impairs the contextual control of response conflict in rats. *Eur. J. Neurosci.* 25 559–566. 10.1111/j.1460-9568.2006.05295.x 17284198

[B136] Marsicano G. Wotjak C. T. Azad S. C. Bisogno T. Rammes G. Cascio M. G. (2002). The endogenous cannabinoid system controls extinction of aversive memories. *Nature* 418 530–534. 10.1038/nature00839 12152079

[B137] Martin M. Ledent C. Parmentier M. Maldonado R. Valverde O. (2002). Involvement of CB1 cannabinoid receptors in emotional behaviour. *Psychopharmacology* 159 379–387. 10.1007/s00213-001-0946-5 11823890

[B138] Matar M. A. Zohar J. Kaplan Z. Cohen H. (2009). Alprazolam treatment immediately after stress exposure interferes with the normal HPA-stress response and increases vulnerability to subsequent stress in an animal model of PTSD. *Eur. Neuropsychopharmacol.* 19 283–295. 10.1016/j.euroneuro.2008.12.004 19167197

[B139] McDonald A. J. (1982). Neurons of the lateral and basolateral amygdaloid nuclei: A Golgi study in the rat. *J. Comp. Neurol.* 212 293–312. 10.1002/cne.902120307 6185547

[B140] McDonald A. J. Augustine J. R. (1993). Localization of GABA-like immunoreactivity in the monkey amygdala. *Neuroscience* 52 281–294. 10.1016/0306-4522(93)90156-a 8450947

[B141] McLaughlin R. J. Hill M. N. Gorzalka B. B. (2014). A critical role for prefrontocortical endocannabinoid signaling in the regulation of stress and emotional behavior. *Neurosci. Biobehav. Rev.* 42 116–131. 10.1016/j.neubiorev.2014.02.006 24582908

[B142] Mechoulam R. Parker L. A. (2013). The endocannabinoid system and the brain. *Annu. Rev. Psychol.* 64 21–47. 10.1146/annurev-psych-113011-143739 22804774

[B143] Medina J. H. Novas M. L. Wolfman C. N. Levi de Stein M. De Robertis E. (1983). Benzodiazepine receptors in rat cerebral cortex and hippocampus undergo rapid and reversible changes after acute stress. *Neuroscience* 9 331–335. 10.1016/0306-4522(83)90298-1 6308509

[B144] Megahed T. Hattiangady B. Shuai B. Shetty A. K. (2015). Parvalbumin and neuropeptide Y expressing hippocampal GABA-ergic inhibitory interneuron numbers decline in a model of Gulf War illness. *Front. Cell. Neurosci.* 8:447. 10.3389/fncel.2014.00447 25620912 PMC4288040

[B145] Meiser-Stedman R. Dalgleish T. Glucksman E. Yule W. Smith P. (2009). Maladaptive cognitive appraisals mediate the evolution of posttraumatic stress reactions: A 6-month follow-up of child and adolescent assault and motor vehicle accident survivors. *J. Abnorm. Psychol.* 118 778–787. 10.1037/a0016945 19899847

[B146] Merali Z. Du L. Hrdina P. Palkovits M. Faludi G. Poulter M. O. (2004). Dysregulation in the suicide brain: mRNA expression of corticotropin-releasing hormone receptors and GABA(A) receptor subunits in frontal cortical brain region. *J. Neurosci.* 24 1478–1485. 10.1523/JNEUROSCI.4734-03.2004 14960621 PMC6730322

[B147] Meyerhoff D. J. Mon A. Metzler T. Neylan T. C. (2014). Cortical gamma-aminobutyric acid and glutamate in posttraumatic stress disorder and their relationships to self-reported sleep quality. *Sleep* 37 893–900. 10.5665/sleep.3654 24790267 PMC3985106

[B148] Michels L. Schulte-Vels T. Schick M. O’Gorman R. L. Zeffiro T. Hasler G. (2014). Prefrontal GABA and glutathione imbalance in posttraumatic stress disorder: Preliminary findings. *Psychiatry Res.* 224 288–295. 10.1016/j.pscychresns.2014.09.007 25448399

[B149] Mikkelsen J. D. Bundzikova J. Larsen M. H. Hansen H. H. Kiss A. (2008). GABA regulates the rat hypothalamic-pituitary-adrenocortical axis via different GABA-A receptor alpha-subtypes. *Ann. N. Y. Acad. Sci.* 1148 384–392. 10.1196/annals.1410.044 19120132

[B150] Milad M. R. Pitman R. K. Ellis C. B. Gold A. L. Shin L. M. Lasko N. B. (2009). Neurobiological basis of failure to recall extinction memory in posttraumatic stress disorder. *Biol. Psychiatry* 66 1075–1082. 10.1016/j.biopsych.2009.06.026 19748076 PMC2787650

[B151] Möller A. T. Bäckström T. Nyberg S. Söndergaard H. P. Helström L. (2016). Women with PTSD have a changed sensitivity to GABA-A receptor active substances. *Psychopharmacology* 233 2025–2033. 10.1007/s00213-014-3776-y 25345735

[B152] Morales M. Wang S. D. Diaz-Ruiz O. Jho D. H. (2004). Cannabinoid CB1 receptor and serotonin 3 receptor subunit A (5-HT3A) are co-expressed in GABA neurons in the rat telencephalon. *J. Comp. Neurol.* 468 205–216. 10.1002/cne.10968 14648680

[B153] Morgan M. A. LeDoux J. E. (1995). Differential contribution of dorsal and ventral medial prefrontal cortex to the acquisition and extinction of conditioned fear in rats. *Behav. Neurosci.* 109 681–688. 10.1037//0735-7044.109.4.681 7576212

[B154] Neumeister A. Normandin M. D. Pietrzak R. H. Piomelli D. Zheng M. Q. Gujarro-Anton A. (2013). Elevated brain cannabinoid CB1 receptor availability in post-traumatic stress disorder: a positron emission tomography study. *Mol. Psychiatry* 18 1034–1040. 10.1038/mp.2013.61 23670490 PMC3752332

[B155] Neuwirth L. S. Verrengia M. T. Harikinish-Murrary Z. I. Orens J. E. Lopez O. E. (2022). Under or absent reporting of light stimuli in testing of anxiety-like behaviors in rodents: the need for standardization. *Front. Mol. Neurosci.* 15:912146. 10.3389/fnmol.2022.912146 36061362 PMC9428565

[B156] Olson V. G. Rockett H. R. Reh R. K. Redila V. A. Tran P. M. Venkov H. A. (2011). The role of norepinephrine in differential response to stress in an animal model of posttraumatic stress disorder. *Biol. Psychiatry* 70 441–448. 10.1016/j.biopsych.2010.11.029 21251647 PMC3740168

[B157] Orchinik M. Weiland N. G. McEwen B. S. (1995). Chronic exposure to stress levels of corticosterone alters GABAA receptor subunit mRNA levels in rat hippocampus. *Brain Res. Mol. Brain Res.* 34 29–37. 10.1016/0169-328x(95)00118-c 8750858

[B158] Orr S. P. Lasko N. B. Metzger L. J. Pitman R. K. (1997). Physiologic responses to non-startling tones in Vietnam veterans with post-traumatic stress disorder. *Psychiatry Res.* 73 103–107. 10.1016/s0165-1781(97)00110-8 9463843

[B159] Orr S. P. Metzger L. J. Lasko N. B. Macklin M. L. Hu F. B. Shalev A. Y. (2003). Physiologic responses to sudden, loud tones in monozygotic twins discordant for combat exposure: Association with posttraumatic stress disorder. *Arch. Gene. Psychiatry* 60 283–288. 10.1001/archpsyc.60.3.283 12622661

[B160] Ouellet-Morin I. Odgers C. L. Danese A. Bowes L. Shakoor S. Papadopoulos A. S. (2011). Blunted cortisol responses to stress signal social and behavioral problems among maltreated/bullied 12-year-old children. *Biol. Psychiatry* 70 1016–1023. 10.1016/j.biopsych.2011.06.017 21839988 PMC3816750

[B161] Pape H. C. Pare D. (2010). Plastic synaptic networks of the amygdala for the acquisition, expression, and extinction of conditioned fear. *Physiol. Rev.* 90 419–463. 10.1152/physrev.00037.2009 20393190 PMC2856122

[B162] Parsons R. Ressler K. (2013). Implications of memory modulation for post-traumatic stress and fear disorders. *Nat. Neurosci.* 16 146–153. 10.1038/nn.3296 23354388 PMC3752300

[B163] Passie T. Emrich H. M. Karst M. Brandt S. D. Halpern J. H. (2012). Mitigation of post-traumatic stress symptoms by *Cannabis resin*: A review of the clinical and neurobiological evidence. *Drug Test. Anal.* 4 649–659. 10.1002/dta.1377 22736575

[B164] Petroff O. A. (2002). GABA and glutamate in the human brain. *Neuroscientist* 8 562–573. 10.1177/1073858402238515 12467378

[B165] Pham X. Sun C. Chen X. van den Oord E. J. Neale M. C. Kendler K. S. (2009). Association study between GABA receptor genes and anxiety spectrum disorders. *Depression Anxiety* 26 998–1003. 10.1002/da.20628 19842164 PMC2783721

[B166] Piomelli D. (2003). The molecular logic of endocannabinoid signalling. *Nat. Rev. Neurosci.* 4 873–884. 10.1038/nrn1247 14595399

[B167] Pitman R. K. Rasmusson A. M. Koenen K. C. Shin L. M. Orr S. P. Gilbertson M. W. (2012). Biological studies of post-traumatic stress disorder. *Nat. Rev. Neurosci.* 13 769–787. 10.1038/nrn3339 23047775 PMC4951157

[B168] Pochwat B. Nowak G. Szewczyk B. (2016). Brain glutamic acid decarboxylase-67kDa alterations induced by magnesium treatment in olfactory bulbectomy and chronic mild stress models in rats. *Pharmacol. Rep.* 68 881–885. 10.1016/j.pharep.2016.04.011 27351943

[B169] Pole N. (2007). The psychophysiology of posttraumatic stress disorder: A meta-analysis. *Psychol. Bull.* 133 725–746. 10.1037/0033-2909.133.5.725 17723027

[B170] Pole N. Neylan T. C. Otte C. Henn-Hasse C. Metzler T. J. Marmar C. R. (2009). Prospective prediction of posttraumatic stress disorder symptoms using fear potentiated auditory startle responses. *Biol. Psychiatry* 65 235–240. 10.1016/j.biopsych.2008.07.015 18722593 PMC2647968

[B171] Pollack M. H. Jensen J. E. Simon N. M. Kaufman R. E. Renshaw P. F. (2008). High-field MRS study of GABA, glutamate and glutamine in social anxiety disorder: Response to treatment with levetiracetam. *Prog. Neuro Psychopharmacol. Biol. Psychiatry* 32 739–743. 10.1016/j.pnpbp.2007.11.023 18206286

[B172] Poulter M. O. Du L. Weaver I. Palkovits M. Faludi G. Merali Z. (2008). GABAA receptor promoter hypermethylation in suicide brain: Implications for the involvement of epigenetic processes. *Biol. Psychiatry* 64 645–652. 10.1016/j.biopsych.2008.05.028 18639864

[B173] Quirk G. J. Russo G. K. Barron J. L. Lebron K. (2000). The role of ventromedial prefrontal cortex in the recovery of extinguished fear. *J. Neurosci.* 20 6225–6231. 10.1523/JNEUROSCI.20-16-06225.2000 10934272 PMC6772571

[B174] Randall P. K. Bremner J. D. Krystal J. H. Nagy L. M. Heninger G. R. Nicolaou A. L. (1995). Effects of the benzodiazepine antagonist flumazenil in PTSD. *Biol. Psychiatry* 38 319–324. 10.1016/0006-3223(94)00306-n 7495926

[B175] Rasmusson A. M. Pineles S. L. (2018). Neurotransmitter, peptide, and steroid hormone abnormalities in PTSD: Biological endophenotypes relevant to treatment. *Curr. Psychiatry Rep.* 20:52. 10.1007/s11920-018-0908-9 30019147

[B176] Rasmusson A. M. Pinna G. Paliwal P. Weisman D. Gottschalk C. Charney D. (2006). Decreased cerebrospinal fluid allopregnanolone levels in women with posttraumatic stress disorder. *Biol. Psychiatry* 60 704–713. 10.1016/j.biopsych.2006.03.026 16934764

[B177] Rauch S. L. Shin L. M. Phelps E. A. (2006). Neurocircuitry models of posttraumatic stress disorder and extinction: Human neuroimaging research–Past, present, and future. *Biol. Psychiatry* 60 376–382. 10.1016/j.biopsych.2006.06.004 16919525

[B178] Raybuck J. D. Lattal K. M. (2011). Double dissociation of amygdala and hippocampal contributions to trace and delay fear conditioning. *PLoS One* 6:e15982. 10.1371/journal.pone.0015982 21283812 PMC3023765

[B179] Raybuck J. D. Lattal K. M. (2014). Differential effects of dorsal hippocampal inactivation on expression of recent and remote drug and fear memory. *Neurosci. Lett.* 569 1–5. 10.1016/j.neulet.2014.02.063 24686177 PMC4067241

[B180] Romeo E. Ströhle A. Spalletta G. di Michele F. Hermann B. Holsboer F. (1998). Effects of antidepressant treatment on neuroactive steroids in major depression. *Am. J. Psychiatry* 155 910–913. 10.1176/ajp.155.7.910 9659856

[B181] Roohbakhsh A. Keshavarz S. Hasanein P. Rezvani M. E. Moghaddam A. H. (2009). Role of endocannabinoid system in the ventral hippocampus of rats in the modulation of anxiety-like behaviours. *Basic Clin. Pharmacol. Toxicol.* 105 333–338. 10.1111/j.1742-7843.2009.00449.x 19614892

[B182] Rosso I. M. Weiner M. R. Crowley D. J. Silveri M. M. Rauch S. L. Jensen J. E. (2014). Insula and anterior cingulate GABA levels in posttraumatic stress disorder: Preliminary findings using magnetic resonance spectroscopy. *Depression Anxiety* 31 115–123. 10.1002/da.22155 23861191 PMC3894264

[B183] Rudolph U. Möhler H. (2004). Analysis of GABAA receptor function and dissection of the pharmacology of benzodiazepines and general anesthetics through mouse genetics. *Annu. Rev. Pharmacol. Toxicol.* 44 475–498. 10.1146/annurev.pharmtox.44.101802.121429 14744255

[B184] Rudolph U. Crestani F. Benke D. Brünig I. Benson J. A. Fritschy J. M. (1999). Benzodiazepine actions mediated by specific gamma-aminobutyric acid(A) receptor subtypes. *Nature* 401 796–800. 10.1038/44579 10548105

[B185] Ruehle S. Rey A. A. Remmers F. Lutz B. (2012). The endocannabinoid system in anxiety, fear memory and habituation. *J. Psychopharmacol. (Oxford, England)* 26 23–39. 10.1177/0269881111408958 21768162 PMC3267552

[B186] Sah P. Faber E. S. Lopez De Armentia M. Power J. (2003). The amygdaloid complex: Anatomy and physiology. *Physiol. Rev.* 83 803–834. 10.1152/physrev.00002.2003 12843409

[B187] Sah P. Westbrook R. F. Lüthi A. (2008). Fear conditioning and long-term potentiation in the amygdala: What really is the connection? *Ann. N. Y. Acad. Sci.* 1129 88–95. 10.1196/annals.1417.020 18591471

[B188] Sakaguchi M. Kim K. Yu L. M. Hashikawa Y. Sekine Y. Okumura Y. (2015). Inhibiting the activity of CA1 hippocampal neurons prevents the recall of contextual fear memory in inducible ArchT transgenic mice. *PLoS One* 10:e0130163. 10.1371/journal.pone.0130163 26075894 PMC4468217

[B189] Sanacora G. Gueorguieva R. Epperson C. N. Wu Y. T. Appel M. Rothman D. L. (2004). Subtype-specific alterations of gamma-aminobutyric acid and glutamate in patients with major depression. *Arch. Gen. Psychiatry* 61 705–713. 10.1001/archpsyc.61.7.705 15237082

[B190] Sangha S. Narayanan R. T. Bergado-Acosta J. R. Stork O. Seidenbecher T. Pape H. C. (2009). Deficiency of the 65 kDa isoform of glutamic acid decarboxylase impairs extinction of cued but not contextual fear memory. *J. Neurosci.* 29 15713–15720. 10.1523/JNEUROSCI.2620-09.2009 20016086 PMC6666166

[B191] Schneider B. L. Ghoddoussi F. Charlton J. L. Kohler R. J. Galloway M. P. Perrine S. A. (2016). Increased cortical gamma-aminobutyric acid precedes incomplete extinction of conditioned fear and increased hippocampal excitatory tone in a mouse model of mild traumatic brain injury. *J. Neurotrauma* 33 1614–1624. 10.1089/neu.2015.4190 26529240

[B192] Schüle C. Nothdurfter C. Rupprecht R. (2014). The role of allopregnanolone in depression and anxiety. *Prog. Neurobiol.* 113 79–87. 10.1016/j.pneurobio.2013.09.003 24215796

[B193] Schwartz T. L. Nihalani N. (2006). Tiagabine in anxiety disorders. *Expert Opin. Pharmacother.* 7 1977–1987. 10.1517/14656566.7.14.1977 17020423

[B194] Sequeira A. Mamdani F. Ernst C. Vawter M. P. Bunney W. E. Lebel V. (2009). Global brain gene expression analysis links glutamatergic and GABAergic alterations to suicide and major depression. *PLoS One* 4:e6585. 10.1371/journal.pone.0006585 19668376 PMC2719799

[B195] Sheth C. Prescot A. P. Legarreta M. Renshaw P. F. McGlade E. Yurgelun-Todd D. (2019). Reduced gamma-amino butyric acid (GABA) and glutamine in the anterior cingulate cortex (ACC) of veterans exposed to trauma. *J. Affect. Disord.* 248 166–174. 10.1016/j.jad.2019.01.037 30735853

[B196] Shin L. M. Liberzon I. (2010). The neurocircuitry of fear, stress, and anxiety disorders. *Neuropsychopharmacology* 35 169–191. 10.1038/npp.2009.83 19625997 PMC3055419

[B197] Sierra-Mercado D. Padilla-Coreano N. Quirk G. J. (2011). Dissociable roles of prelimbic and infralimbic cortices, ventral hippocampus, and basolateral amygdala in the expression and extinction of conditioned fear. *Neuropsychopharmacology* 36 529–538. 10.1038/npp.2010.184 20962768 PMC3005957

[B198] Sigurdsson T. Doyère V. Cain C. K. LeDoux J. E. (2007). Long-term potentiation in the amygdala: A cellular mechanism of fear learning and memory. *Neuropharmacology* 52 215–227. 10.1016/j.neuropharm.2006.06.022 16919687

[B199] Singewald N. Schmuckermair C. Whittle N. Holmes A. Ressler K. J. (2015). Pharmacology of cognitive enhancers for exposure-based therapy of fear, anxiety and trauma-related disorders. *Pharmacol. Ther.* 149 150–190. 10.1016/j.pharmthera.2014.12.004 25550231 PMC4380664

[B200] Smoller J. (2016). The genetics of stress-related disorders: PTSD, depression, and anxiety disorders. *Neuropsychopharmacology* 41 297–319. 10.1038/npp.2015.266 26321314 PMC4677147

[B201] Song C. Zhang W. H. Wang X. H. Zhang J. Y. Tian X. L. Yin X. P. (2017). Acute stress enhances the glutamatergic transmission onto basoamygdala neurons embedded in distinct microcircuits. *Mol. Brain* 10:3. 10.1186/s13041-016-0283-6 28069030 PMC5223467

[B202] Spivak B. Maayan R. Kotler M. Mester R. Gil-Ad I. Shtaif B. (2000). Elevated circulatory level of GABA(A)–antagonistic neurosteroids in patients with combat-related post-traumatic stress disorder. *Psychol. Med.* 30 1227–1231. 10.1017/s0033291799002731 12027057

[B203] Stachowicz K. (2018). The role of DSCAM in the regulation of synaptic plasticity: Possible involvement in neuropsychiatric disorders. *Acta Neurobiol. Exp.* 78 210–219. 30295678

[B204] Suendermann O. Ehlers A. Boellinghaus I. Gamer M. Glucksman E. (2010). Early heart rate responses to standardized trauma-related pictures predict posttraumatic stress disorder: A prospective study. *Psychosom. Med.* 72 301–308. 10.1097/PSY.0b013e3181d07db8 20124426 PMC2865997

[B205] Sun K. Fan J. Han J. (2015). Ameliorating effects of traditional Chinese medicine preparation, Chinese materia medica and active compounds on ischemia/reperfusion-induced cerebral microcirculatory disturbances and neuron damage. *Acta Pharm. Sin. B* 5 8–24. 10.1016/j.apsb.2014.11.002 26579420 PMC4629119

[B206] Sun X. Song Z. Si Y. Wang J. H. (2018). microRNA and mRNA profiles in ventral tegmental area relevant to stress-induced depression and resilience. *Prog. Neuro Psychopharmacol. Biol. Psychiatry* 86 150–165. 10.1016/j.pnpbp.2018.05.023 29864451

[B207] Sutherland S. M. Davidson J. R. (1994). Pharmacotherapy for post-traumatic stress disorder. *Psychiatric Clin. North Am.* 17 409–423. 7937367

[B208] Szabó G. G. Lenkey N. Holderith N. Andrási T. Nusser Z. Hájos N. (2014). Presynaptic calcium channel inhibition underlies CB_1_ cannabinoid receptor-mediated suppression of GABA release. *J. Neurosci.* 34 7958–7963. 10.1523/JNEUROSCI.0247-14.2014 24899717 PMC6608264

[B209] Taylor F. B. (2003). Tiagabine for posttraumatic stress disorder: A case series of 7 women. *J. Clin. Psychiatry* 64 1421–1425. 10.4088/jcp.v64n1204 14728102

[B210] Tovote P. Fadok J. P. Lüthi A. (2015). Neuronal circuits for fear and anxiety. *Nat. Rev. Neurosci.* 16 317–331. 10.1038/nrn3945 25991441

[B211] Trezza V. Campolongo P. (2013). The endocannabinoid system as a possible target to treat both the cognitive and emotional features of post-traumatic stress disorder (PTSD). *Front. Behav. Neurosci.* 7:100. 10.3389/fnbeh.2013.00100 23950739 PMC3739026

[B212] Twining R. C. Lepak K. Kirry A. J. Gilmartin M. R. (2020). Ventral hippocampal input to the prelimbic cortex dissociates the context from the cue association in trace fear memory. *J. Neurosci.* 40 3217–3230. 10.1523/JNEUROSCI.1453-19.2020 32188770 PMC7159889

[B213] Uniyal A. Singh R. Akhtar A. Bansal Y. Kuhad A. Sah S. P. (2019). Co-treatment of piracetam with risperidone rescued extinction deficits in experimental paradigms of post-traumatic stress disorder by restoring the physiological alterations in cortex and hippocampus. *Pharmacol. Biochem. Behav.* 185:172763. 10.1016/j.pbb.2019.172763 31445955

[B214] Uzunova V. Sheline Y. Davis J. M. Rasmusson A. Uzunov D. P. Costa E. (1998). Increase in the cerebrospinal fluid content of neurosteroids in patients with unipolar major depression who are receiving fluoxetine or fluvoxamine. *Proc. Natl. Acad. Sci. U.S.A.* 95 3239–3244. 10.1073/pnas.95.6.3239 9501247 PMC19726

[B215] Vaiva G. Boss V. Ducrocq F. Fontaine M. Devos P. Brunet A. (2006). Relationship between posttrauma GABA plasma levels and PTSD at 1-year follow-up. *Am. J. Psychiatry* 163 1446–1448. 10.1176/ajp.2006.163.8.1446 16877663

[B216] Vaiva G. Thomas P. Ducrocq F. Fontaine M. Boss V. Devos P. (2004). Low posttrauma GABA plasma levels as a predictive factor in the development of acute posttraumatic stress disorder. *Biol. Psychiatry* 55 250–254. 10.1016/j.biopsych.2003.08.009 14744465

[B217] Valentinuzzi V. S. Kolker D. E. Vitaterna M. H. Shimomura K. Whiteley A. Low-Zeddies S. (1998). Automated measurement of mouse freezing behavior and its use for quantitative trait locus analysis of contextual fear conditioning in (BALB/cJ x C57BL/6J)F2 mice. *Learn. Mem. (Cold Spring Harbor, N.Y.)* 5 391–403. 10454363 PMC311259

[B218] Vasterling J. Brewin C. R. (2005). *Neuropsychology of PTSD : Biological, cognitive, and clinical perspectives.* New York, NY: Guilford Press.

[B219] Verkuyl J. M. Joëls M. (2003). Effect of adrenalectomy on miniature inhibitory postsynaptic currents in the paraventricular nucleus of the hypothalamus. *J. Neurophysiol.* 89 237–245. 10.1152/jn.00401.2002 12522175

[B220] Verkuyl J. M. Karst H. Joëls M. (2005). GABAergic transmission in the rat paraventricular nucleus of the hypothalamus is suppressed by corticosterone and stress. *Eur. J. Neurosci.* 21 113–121. 10.1111/j.1460-9568.2004.03846.x 15654848

[B221] Viola J. Ditzler T. Batzer W. Harazin J. Adams D. Lettich L. (1997). Pharmacological management of post-traumatic stress disorder: clinical summary of a five-year retrospective study, 1990-1995. *Military Med.* 162 616–619. 9290298

[B222] Wilensky A. E. Schafe G. E. Kristensen M. P. LeDoux J. E. (2006). Rethinking the fear circuit: the central nucleus of the amygdala is required for the acquisition, consolidation, and expression of Pavlovian fear conditioning. *J. Neurosci.* 26 12387–12396. 10.1523/JNEUROSCI.4316-06.2006 17135400 PMC6674909

[B223] Wilson J. Keane T. M. (1997). *Assessing psychological trauma and PTSD.* New York, NY: Guilford Press.

[B224] Wolfe J. Chrestman K. R. Ouimette P. C. Kaloupek D. Harley R. M. Bucsela M. (2000). Trauma-related psychophysiological reactivity in women exposed to war-zone stress. *J. Clin. Psychol.* 56 1371–1379. 10.1002/1097-4679(200010)56:10<1371::AID-JCLP8<3.0.CO;2-X 11051064

[B225] Xu W. Südhof T. C. (2013). A neural circuit for memory specificity and generalization. *Science (New York, N.Y.)* 339 1290–1295. 10.1126/science.1229534 23493706 PMC3651700

[B226] Zannas A. Provençal N. Binder E. (2015). Epigenetics of posttraumatic stress disorder: Current evidence, challenges, and future directions. *Biol. Psychiatry* 78 327–335. 10.1016/j.biopsych.2015.04.003 25979620

[B227] Zhang D. Pan Z. H. Awobuluyi M. Lipton S. A. (2001). Structure and function of GABA(C) receptors: A comparison of native versus recombinant receptors. *Trends Pharmacol. Sci.* 22 121–132. 10.1016/s0165-6147(00)01625-4 11239575

[B228] Zhang S. Cranney J. (2008). The role of GABA and anxiety in the reconsolidation of conditioned fear. *Behav. Neurosci.* 122 1295–1305. 10.1037/a0013273 19045949

