# Corrigendum: Involvement of the GABAergic system in PTSD and its therapeutic significance

**DOI:** 10.3389/fnmol.2023.1158825

**Published:** 2023-02-20

**Authors:** Junhui Huang, Fei Xu, Liping Yang, Lina Tuolihong, Xiaoyu Wang, Zibo Du, Yiqi Zhang, Xuanlin Yin, Yingjun Li, Kangrong Lu, Wanshan Wang

**Affiliations:** ^1^Southern Medical University, Guangzhou, China; ^2^Department of Applied Psychology of School of Public Health, Southern Medical University, Guangzhou, China; ^3^Department of Basic Medical of Basic Medical College, Southern Medical University, Guangzhou, China; ^4^Eight-Year Master's and Doctoral Program in Clinical Medicine of the First Clinical Medical College, Southern Medical University, Guangzhou, China; ^5^Department of Medical Laboratory Science, School of Laboratory Medicine and Biotechnology, Southern Medical University, Guangzhou, China; ^6^Guangdong Provincial Key Laboratory of Construction and Detection in Tissue Engineering, Southern Medical University, Guangzhou, China; ^7^Department of Laboratory Animal Center, Southern Medical University, Guangzhou, China

**Keywords:** post-traumatic stress disorder, GABA, fear memory, amygdala, neurotransmitter

In the published article, there was an error in affiliations [1, 2]. Instead of “[^1^Clinical Excellence and Innovation Class of the Second Clinical Medical College, Southern Medical University, Guangzhou, China, ^2^Department of Psychiatry of School of Public Health, Southern Medical University, Guangzhou, China],” it should be “[^1^Southern Medical University, Guangzhou, China].”

The authors apologize for this error and state that this does not change the scientific conclusions of the article in any way. The original article has been updated.

